# Petal abscission in fragrant roses is associated with large scale differential regulation of the abscission zone transcriptome

**DOI:** 10.1038/s41598-020-74144-3

**Published:** 2020-10-14

**Authors:** Priya Singh, Neeraj Bharti, Amar Pal Singh, Siddharth Kaushal Tripathi, Saurabh Prakash Pandey, Abhishek Singh Chauhan, Abhijeet Kulkarni, Aniruddha P. Sane

**Affiliations:** 1 Molecular Biology and Biotechnology, CSIR-National Botanical Research Institute, Lucknow, 226001 India; 2 Academy of Scientific and Innovative Research (AcSIR), Ghaziabad, 201002 India; 3 Bioinformatics Centre, Savitribai Phule Pune University, Pune, 411007 India; 4 High Performance Computing-Medical and Bioinformatics Applications Group, Centre for Development of Advanced Computing, Pune, 411008 India; 5 Present Address: National Institute for Plant Genome Research, New Delhi, 110067 India; 6 Present Address: National Centre for Natural Products Research, School of Pharmacy, University of Mississippi, Oxford, MS 38677 USA

**Keywords:** Computational biology and bioinformatics, Plant sciences

## Abstract

Flowers of fragrant roses such as *Rosa bourboniana* are ethylene-sensitive and undergo rapid petal abscission while hybrid roses show reduced ethylene sensitivity and delayed abscission. To understand the molecular mechanism underlying these differences, a comparative transcriptome of petal abscission zones (AZ) of 0 h and 8 h ethylene-treated flowers from *R. bourboniana* was performed. Differential regulation of 3700 genes (1518 up, 2182 down) representing 8.5% of the AZ transcriptome was observed between 0 and 8 h ethylene-treated *R. bourboniana* petal AZ. Abscission was associated with large scale up-regulation of the ethylene pathway but prominent suppression of the JA, auxin and light-regulated pathways. Regulatory genes encoding kinases/phosphatases/F-box proteins and transcription factors formed the major group undergoing differential regulation besides genes for transporters, wall modification, defense and phenylpropanoid pathways. Further comparisons with ethylene-treated petals of *R. bourboniana* and 8 h ethylene-treated AZ (*R. hybrida*) identified a core set of 255 genes uniquely regulated by ethylene in *R. bourboniana* AZ. Almost 23% of these encoded regulatory proteins largely conserved with Arabidopsis AZ components. Most of these were up-regulated while an entire set of photosystem genes was prominently down-regulated. The studies provide important information on regulation of petal abscission in roses.

## Introduction

Organ abscission is an important developmental process that regulates the detachment of leaves, flowers, flower parts, fruits etc. from main body during the course of development^1^. Controlled abscission of old, diseased or surplus organs is necessary to conserve resources for developing organs to maintain healthy growth while abscission of fruits and seeds ensures reproductive success and survival through dispersal^1–4^. Although abscission leads to separation of an entire organ from a plant, the processes leading to separation are controlled by cells of a small zone, the abscission zone (AZ). The AZ, only a few cell layers thick, shows differential sensitivity to hormones and differential regulation of genes that trigger abscission. Hormones that either promote or inhibit abscission have to be maintained in a fine balance. Ethylene is one of the most important of these in initiating abscission in most plants including trees^5–12^. Mutants of ethylene perception and signaling show delayed organ abscission in *Arabidopsis* and tomato^13–19^. Not surprisingly, components of the ethylene biosynthesis and signal pathway are often expressed in an AZ-specific manner^9,20–25^.

Unlike ethylene, auxin inhibits or delays abscission in leaves, flower and fruits^1, 2^. Components of auxin transport^26^ and signalling including auxin response factors (ARFs)^27–30^ play an important role in abscission. Other hormones like abscisic acid (ABA) and jasmonic acid (JA) also influence abscission although their involvement at the molecular level is much less studied^31–34^.

Although abscission is known in several plants, the key determinants of abscission have primarily been identified in the model plant Arabidopsis where genes like *HAESA* and *HAESA LIKE2* encoding LRR type receptor kinases^35–37^, *IDA*^35,37^, *AGL15*^38^, *AGL18*^39^, MAP kinases *MPK3* and *MPK6*^37^, *NEVERSHED*^40^, *EVERSHED*^41^, *FOREVER YOUNG FLOWER*^42^, have been characterized. In rice, studies on the process of seed shattering have led to identification of *SH4*^43^, *qSH1*^44^, *SHAT1*^45^ and *SH5*^46^, while abscission of tomato has been shown to be controlled by a MADS box gene, *JOINTLESS*^47,48^. In most other plants, regulatory genes controlling abscission remain to be identified.

The availability of microarray at the turn of the century and NGS techniques over the last 10 years has enabled large scale gene expression analysis of even fine tissues such as organ AZ in model plants like *Arabidopsis*^49^ and tomato^50–53^ as well as a few other plants like citrus^12^, apple^54^, melon^55^, olive^56^, litchi^57^, sugarcane^58^. Nevertheless, the difficulties associated with isolation of the abscission zones limit studies on organ abscission in most plants even by NGS techniques.

Rose is an important flower in the floriculture and fragrance industries. The fragrant variety of rose (such as *Rosa bourboniana*) is popular but sensitive to ethylene. It has a short vase life of 1 to 2 days post-pollination that decreases its commercial value. In contrast, the non-fragrant *R. hybrida* is less sensitive to ethylene and has greater vase life of several days. These differences are partly associated with differences in sensitivities of the ethylene pathway^25^ which in turn affect expression of cell wall modifying genes and programmed cell death-like processes^11, 59–63^. Nevertheless, the molecular determinants that are responsible for regulation of petal abscission in fragrant and non-fragrant roses remain unclear and need to be identified to increase flower life in roses.

In the present study we show, through transcriptional profiling of petal AZ of ethylene-treated flowers of *R. bourboniana* and *R. hybrida*, a prominent alteration in expression of a large number of regulatory genes that include transcription factors, kinases/phosphatases and F-box proteins besides many other pathways. We also show the activation of the ethylene pathway but a suppression of the JA, auxin and light-governed pathways during the course of abscission. The study provides important information on abscission regulation in roses that could be used for improvement of ornamentals.

## Results

### Illumina-based sequencing and assembly reveals large scale transcriptomic changes during ethylene-induced petal abscission in roses

To understand the processes governing ethylene-induced petal abscission in rose, a comparison of the transcriptome data was carried out between 0 h (ethylene-untreated) and 8 h ethylene-treated (representing the mid-point of abscission) petal AZs from *R. bourboniana* on an Illumina Hi-Seq2000 in three independent replicates. Further comparisons were also carried out between 8 h ethylene-treated *R. bourboniana* petal AZ with ethylene-treated whole petals and with 8 h ethylene-treated petal AZs of *R. hybrida.* A total of 32,961 genes were found expressing in the rose petal AZ transcriptome out of 43,301 genes predicted in the genome. Very little variation in expression was observed in FPKM plot comparisons of biological replicates in contrast to comparisons of control and experimental samples indicating high reproducibility of the experimental data set (Fig. S1). Following assembly, a comparative analysis was performed to identify differentially expressed genes (DEGs) in abscission that were defined as significantly up- or down-regulated based on a log_2_-fold change (|FC|≥ 1 and ≤  − 1 respectively) and *P* value ≤ 0.05.

**Figure 1:**
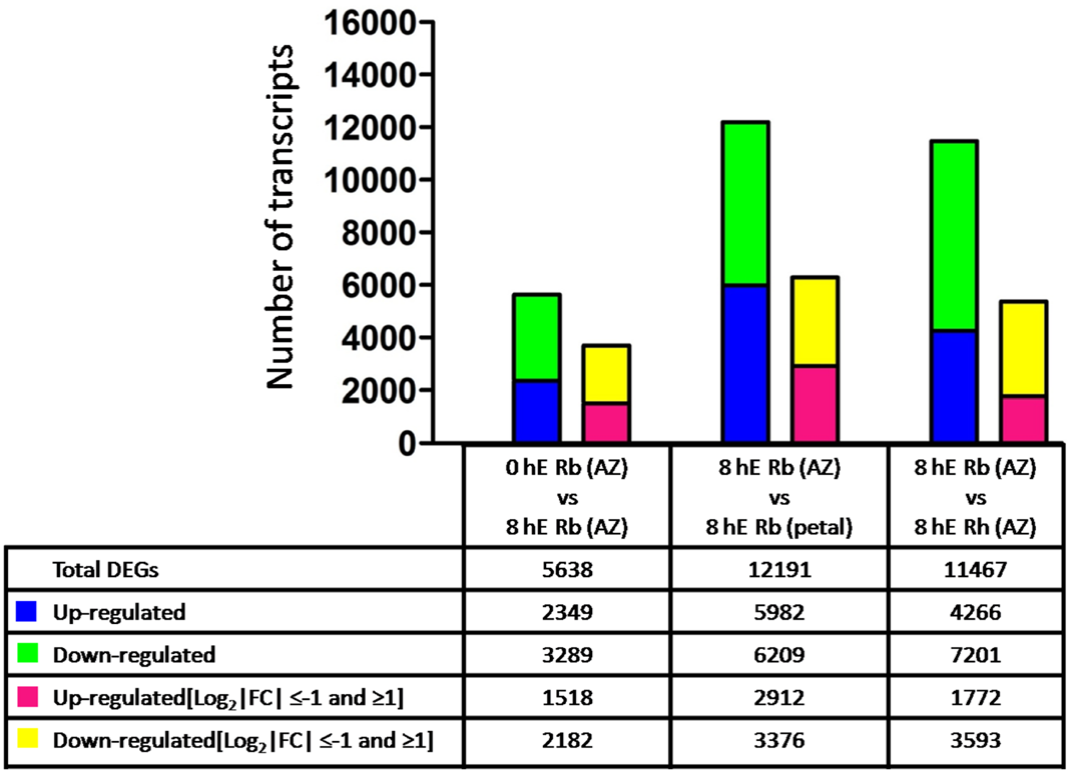
Summary of differentially expressed genes obtained from all comparisons. DEGs obtained from pair-wise comparisons of 0 h versus 8 h ethylene-treated petal AZ of *R. bourboniana* (labeled as 0 hE Rb AZ and 8 hE Rb AZ respectively), 8 h ethylene-treated petal AZ versus petal of *R. bourboniana* (8 hE Rb petal) and 8 h ethylene-treated petal AZ (*R. bourboniana*) versus 8 h ethylene-treated petal AZ of *R. hybrida* (8 hE Rh AZ) transcriptomes with all log_2_FC and (log_2_ |FC|≤ − 1 and ≥ 1) at Q-value < 0.05. Numbers of up- and down-regulated genes are summarized.

A total of 5638 significant DEGs could be identified in the comparison between 0 h versus 8 h ethylene-treated AZ samples of *R. bourboniana* while 12,191 DEGs were seen in the comparison between 8 h ethylene-treated petal AZ versus 8 h ethylene-treated whole petal samples of *R. bourboniana* and 11,467 DEGs in the comparison between 8 h ethylene-treated AZ samples of *R. bourboniana* versus 8 h ethylene-treated AZ samples of *R. hybrida* (Table S2). Of this, a total of 2349, 5982 and 4266 genes were significantly differentially up-regulated and 3289, 6209 and 7201 genes were significantly down-regulated in *R. bourboniana* petal AZ in the 0 h versus 8 h ethylene-treated AZ (*R. bourboniana*), 8 h ethylene-treated petal AZ versus whole petal (*R. bourboniana*) and 8 h ethylene-treated *R. bourboniana* AZ versus *R. hybrida* AZ transcriptome comparisons, respectively. Of the significant DEGs in all comparisons, a further analysis of genes at log_2_FC > 1 and <  − 1 was carried out. A total of 1518, 2912 and 1772 genes were significantly differentially up-regulated and 2182, 3376 and 3593 genes were significantly down-regulated in *R. bourboniana* petal AZ in the 0 h versus 8 h ethylene-treated AZ (*R. bourboniana*), 8 h ethylene-treated AZ versus petal (*R. bourboniana*) and 8 h ethylene-treated *R. bourboniana* AZ versus *R. hybrida* AZ transcriptome comparisons, respectively (Fig. 1). A larger number of genes were down-regulated with the onset of abscission (Fig. 1). These genes (significant at log_2_FC > 1 and log_2_FC <  − 1) were considered for further analysis.

The expression profiles of eight housekeeping genes namely, *ELONGATION FACTOR EF1Α* (JN39925), *UBIQUITIN* (JN39927), *GLYCERALDEHYDE-3-PHOSPHATE DEHYDROGENASE* (JN39920), *SERINE/THREONINE PROTEIN PHOSPHATASE 2A* (JN39924), *SAND* (JN39928), *TUBULIN* (JN39923), *TIP* (JN39921) and *ACTIN* (KF985187)^64,65^ revealed no significant variation in the four transcriptome samples used in the analysis (Fig. S2).

### Abscission is associated with a substantial change in the expression of transcriptional and post-translational regulators

**Figure 2:**
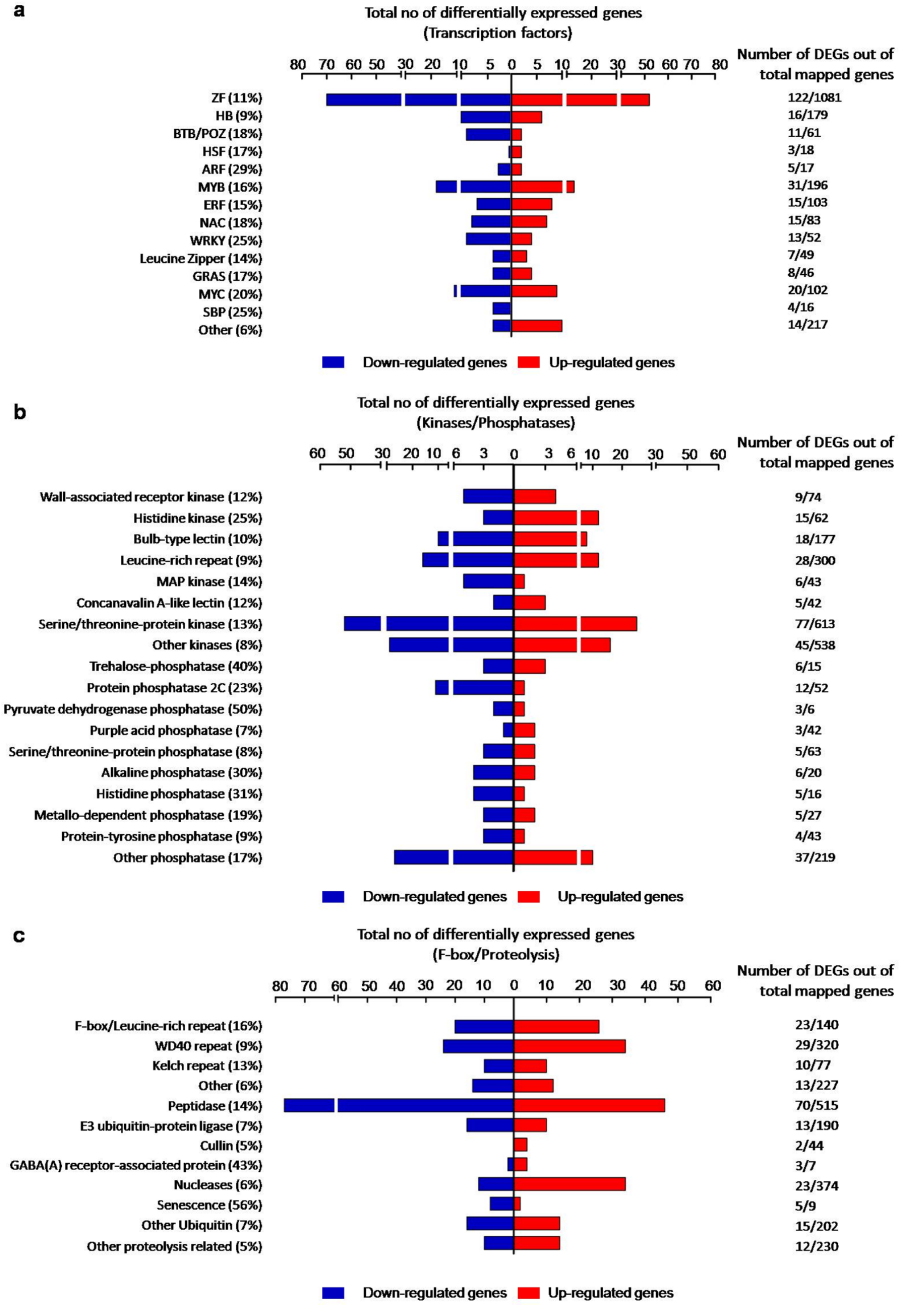
Functional cataloguing showing the proportion of DEGs encoding regulatory proteins in the ethylene-treated *R. bourboniana* petal AZ transcriptome (**a**) transcription factors, (**b**) kinases and phosphatases, (**c**) F-box and proteolysis components. Analysis was performed at [log_2_|FC|(≤ − 1 and ≥ 1)], Q-value < 0.05. Percentage (%) reflects the percentage of DEGs at [log_2_|FC|(≤ − 1 and ≥ 1)] at Q-value < 0.05 out of total mapped genes at any fold change and Q-value.

**Figure 3:**
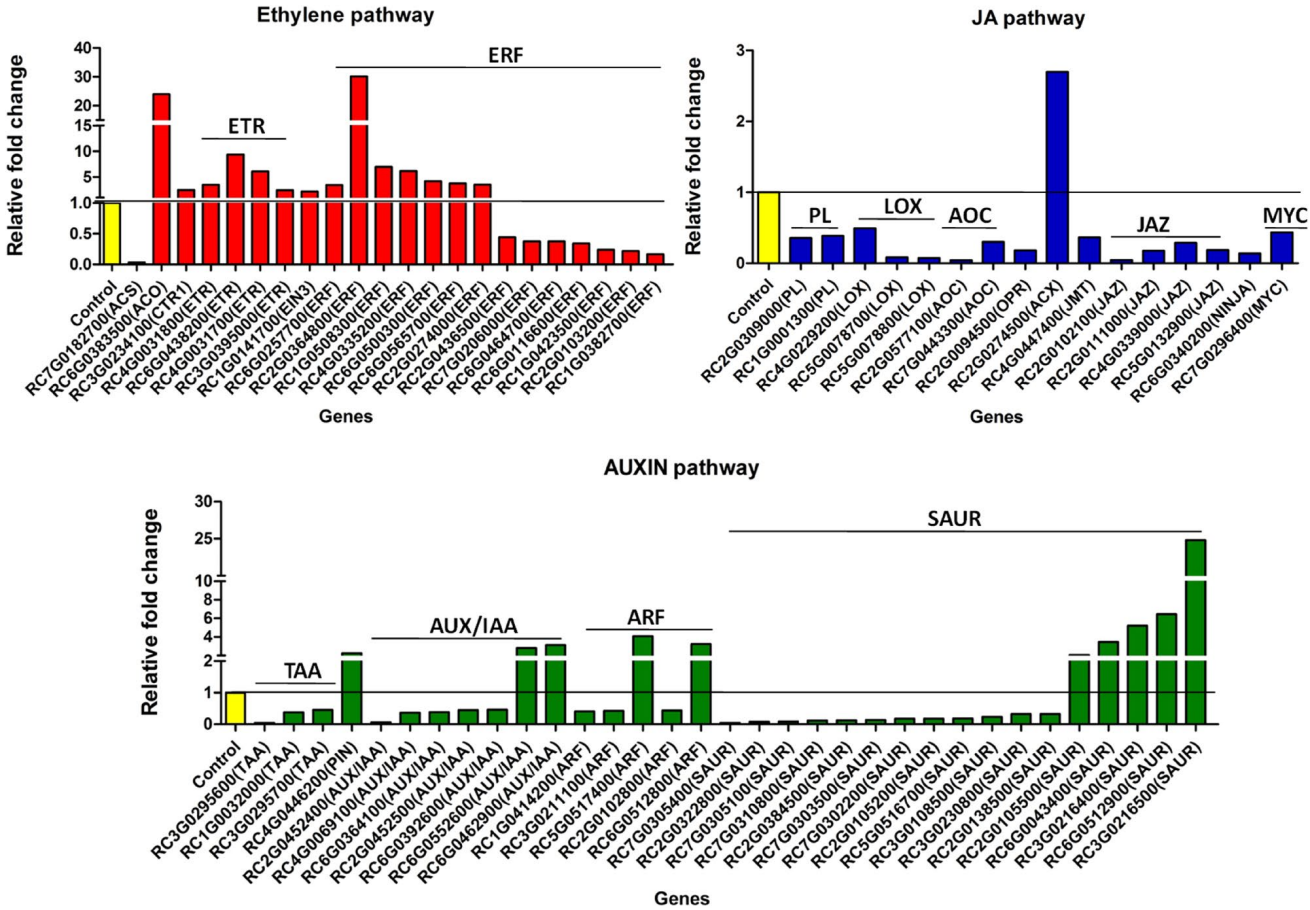
Expression profile of differential AZ genes belonging to ethylene (**a**), JA (**b**) and auxin (**c**) pathways. Bars represent the relative fold expression change after ethylene treatment as calculated from transcriptome data using the expression in all three biological replicates (log_2_ |FC|≤ − 1 and ≥ 1, Q-value < 0.05). Expression of respective genes in controls was taken as one and shown as a black line across genes for comparison.

**Figure 4:**
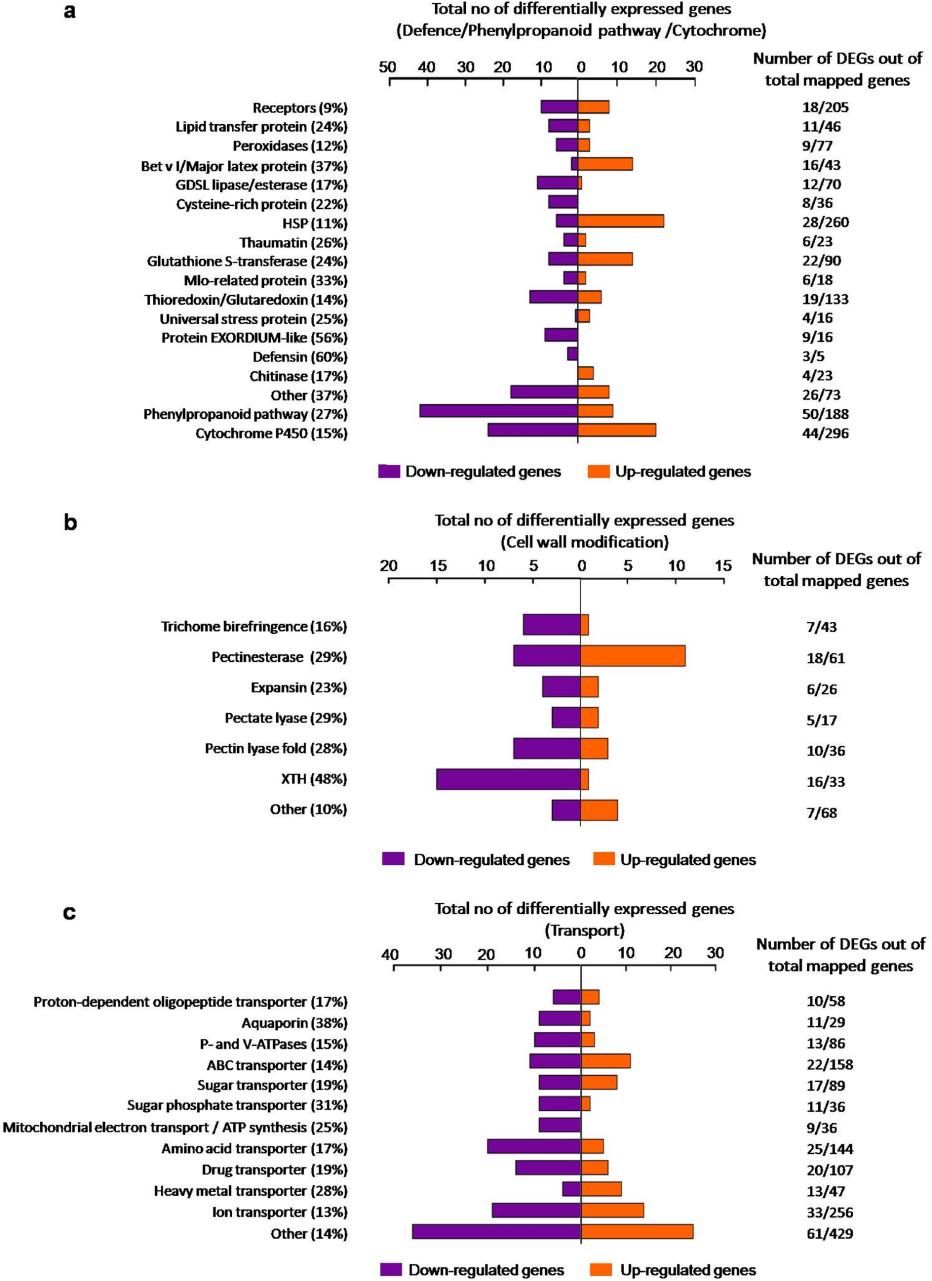
Functional cataloguing showing the proportion of DEGs associated with defense, wall modification and transport (**a**) Defence-related components (**b**) wall modification proteins, (**c**) transporters. Analysis was performed at [log_2_|FC|(≤ − 1 and ≥ 1)], Q-value < 0.05. Percentage (%) reflects the percentage of DEGs at [log_2_|FC|(≤ − 1 and ≥ 1)] at Q-value < 0.05 out of total mapped genes at any fold change and Q-value.

An analysis of all DEGs in the 0 h versus 8 h ethylene-treated petal AZ RNA was next performed to identify important biological pathways affected during ethylene-induced petal abscission. The most prominently affected genes in the transcriptome belonged to the transcription factor families, kinases/phosphatases and F-box protein degradation families, hormone signalling components, transporters, biotic/abiotic stress groups, cell wall modification and carbon metabolism (Figs. 2, 3, 4, Tables S3, S4).

Of the 5638 DEGs, 284 genes (122 up, 162 down) encoding putative transcription factors (TFs) were differentially regulated between the 0 and 8 h ethylene-treated petal AZ samples (Fig. 2a; Table S3). Prominent among the differentially regulated TF groups were zinc finger proteins (52 up, 70 down), MYBs (13 up, 18 down), MYCs (9 up, 11 down), homeobox domain genes (6 up, 10 down), ERFs (8 up, 7 down,), NACs (7 up, 8 down), WRKYs (4 up, 9 down) and BTB/POZ (2 up, 9 down). Other groups included GRAS (4 up, 4 down), Leucine zipper genes (3 up, 4 down), ARFs (2 up, 3 down), SBP (4 down), heat shock factors (HSFs; 2 up, 1 down), MADSs (2 down) and LOBs (2 up).

Post-translations modifications are an important form of regulation of a large number of proteins and enzymes including TFs during development. One of the most prominent modifications includes phosphorylation/dephosphorylation brought about by kinases and phosphatases. Not surprisingly, genes encoding these accounted for 289 DEGs in the rose abscission transcriptome (Fig. 2b; Table S3). Of these, kinases accounted for 203 DEGs, of which 81 were up-regulated while 122 were down-regulated. The majority encoded serine threonine protein kinases (25 up-, 52 down-regulated) and LRR kinases (12 up- and 16 down-regulated). The histidine kinase group (12 up, 3 down) showed an unusually staggered up-regulation and included three ethylene receptors ETR3, ETR7 and ETR8, one cytokinin receptor and three phytochrome genes, *PhyA, PhyB* and *PhyE*. Wall-associated receptor kinases (4 up- and 5 down-regulated), mitogen-activated protein kinases (1 up- and 5 down-regulated) and bulb-type lectin kinases (8 up- and 10 down-regulated) were also abundant. Compared with kinases, phosphatases accounted for a much smaller yet substantial proportion of the DEGs with 86 genes (25 up-regulated, 61 down-regulated). The PP2C group of phosphatases accounted for the majority of genes and were down-regulated (Fig. 2b; Table S3).

In addition, proteins that control turnover of key regulatory proteins in signal transduction pathways and other important cellular processes also play an important role in development. A group of 75 DEGs encoding F-box proteins, involved in proteasomal degradation of important proteins, were observed. These belonged to different groups containing additional domains such as WD repeat (29), LRR (23), Kelch (10) etc. (Fig. 2c). Of the 75, 41 genes were up-regulated while 34 genes were down-regulated in 8 h ethylene-treated petal abscission zones. In addition, 100 genes encoding peptidases and members of the ubiquitin E3 ligase complex (that include cullin and ubiquitin-conjugating enzymes responsible for protein ubiquitylation and degradation) were differentially regulated. Of these, 37 genes were up-regulated while 63 genes were down-regulated (Fig. 2c; Table S3). Amongst both groups, the proteasome components were largely up-regulated. Processes associated with nucleic acid degradation and programmed cell death (PCD) (23 genes), senescence (5 genes) and autophagy (3 genes) were also differentially regulated with most members of nucleic acid degradation being up-regulated during abscission (Fig. 2c; Table S3).

### Phytohormone pathways undergo prominent changes with the onset and progression of abscission

Phytohormones regulate almost every aspect of plant growth and adaptation. They also regulate and are known, in turn, to be regulated in response to abscission. To examine phytohormone involvement during abscission in rose, genes encoding biosynthesis/signaling components of key abscission hormones like ethylene, auxin, jasmonic acid (JA), abscisic acid (ABA), salicylic acid (SA), GA, cytokinin and brassinosteroid were analyzed. Among a total of 111 hormone pathway-related DEGs, the auxin and ethylene signalling pathways were the largest groups with 36 and 24 DEGs, respectively (Table S4). The majority of auxin pathway genes encoding tryptophan synthase, AUX/IAA proteins, auxin response factors and small auxin up RNA were down-regulated while a few genes encoding an auxin efflux carrier (RC4G0446200), two AUX/IAA proteins (RC6G0552600, RC6G0462900), two auxin response factors (RC5G0517400, RC6G0512800) and five small auxin up RNAs (from RC2G0105500 to RC3G0216500) were up-regulated during abscission (Fig. 3c). In contrast, the majority of ethylene pathway genes were up-regulated during abscission. These included 7 genes encoding 1-aminocyclopropane-1-carboxylic acid oxidase (ACO; RC6G0383500), ethylene receptors (ETRs; from RC4G0031800- RC3G0395000,) and signalling components like CONSTITUTIVE TRIPLE RESPONSE (CTR; RC3G0234100) and ETHYLENE INSENSITIVE 3 (EIN3; RC1G0141700). A gene encoding the ethylene biosynthesis enzyme 1-aminocyclopropane-1-carboxylic acid synthase (ACS; RC7G0182700) was down-regulated while 7 of the 14 differentially regulated ERFs were up-regulated and 7 down-regulated (Fig. 3a). Interestingly, genes encoding components of the JA pathway were prominently down-regulated. These included genes encoding homologues of phospholipase A and D (PLA, RC2G0309000; PLD, RC1G0001300), lipoxygenases (LOXs; RC4G0229200, RC5G0078700, RC5G0078800), allene oxide cyclases (AOCs, RC7G0443300, RC2G0577100), acyl-coenzyme A oxidase (RC2G0274500) and an OPDA reductase (OPR, RC2G0094500) involved in JA biosynthesis (Fig. 3b). As an exception, an acyl-coenzyme A oxidase gene (RC2G0274500) was up-regulated. The expression of four JASMONATE-ZIM-DOMAIN PROTEIN genes (JAZs, RC2G0102100, RC2G0111000, RC4G0339000, RC5G0132900), encoding key repressors of the JA-pathway, was down-regulated. One jasmonic acid carboxyl methyltransferase (JMT, RC4G0447400) and one MYC transcription factor homologue (RC7G0296400) that are known to govern jasmonate-regulated plant responses in Arabidopsis were also down-regulated. Among DEGs of the ABA pathway, the expression of the gene encoding the rate-limiting ABA biosynthesis enzyme, 9-cis-epoxycarotenoid dioxygenase (NCED; RC5G0132400) was lower in 8 h ethylene-treated petal AZ. Simultaneously, the expression of genes encoding abscisic acid 8′-hydroxylases (CYP707A; RC6G0562100 and RC6G0114600) that are key ABA catabolism enzymes, was up-regulated. On the other hand, two genes encoding homologues of abscisic aldehyde oxidase (AAO; RC5G0431800) and abscisic acid deficient 4 protein (ABA4; RC7G0271400) possibly involved in ABA biosynthesis and de novo ABA synthesis respectively, were up-regulated. Many protein phosphatase 2Cs (PP2Cs; RC1G0573600, RC5G0571100 and RC6G0083700) which may function as global negative regulators of ABA signalling were down-regulated suggesting that the balance between ABA biosynthesis and catabolism is tightly controlled during rose petal abscission (Fig. S3a). The cytokinin pathway also showed a similar profile with DEGs encoding proteins of cytokinin biosynthesis such as cytokinin synthase (RC4G0245000), cytokinin riboside 5′-monophosphate phosphoribohydrolase (LOG, RC6G0513700), catabolism genes such as cytokinin dehydrogenase (RC6G0597300 and RC1G0050300) and a histidine kinase gene (RC4G0454300) associated with cytokinin signalling all being up-regulated simultaneously. This suggested that the cytokinin pathway is under complex control during abscission (Fig. S3b). For the GA pathway, one of the primary genes involved in GA biosynthesis encoding a GA20 oxidase (RC1G0310000) was up-regulated while another encoding a GA2 oxidase (RC5G0037300) that inactivates GAs, was down-regulated. GIBBERELLIN INSENSITIVE DWARF1 (RC6G0417300), which initiates GA signalling by promoting degradation of the GA-inhibitory DELLA proteins was also up-regulated suggesting a possible increase in the GA response although other members of the pathway were not affected much (Fig. S3c). Some DEGs related to the SA pathway also underwent change during abscission (Fig. S3d).

### Abscission affects the expression of defence and stress pathways and regulates carbon metabolism and transport

A large number of stress and defense-associated pathways were also regulated during abscission. Within stress, those encoding pathogenesis-related proteins (39 genes, encoding chitinases, thaumatin-like proteins, defensins, Toll/interleukin-1 receptor, glutamate receptor) and oxidative stress proteins (55 genes, encoding peroxidases, glutaredoxins, thioredoxins and glutathone S-transferases) were abundant. (Fig. 4a; Table S3). Heat shock proteins, which are also reported to express upon wounding and defence, showed a pattern unlike other groups in that 22/28 HSPs were up-regulated and only 6 were down-regulated during abscission (Fig. 4a; Table S3). Likewise, 17/20 DEGs in abiotic stress were up-regulated while 23/29 DEGs associated with biotic stress were down-regulated.

The phenylpropanoid pathway, involved in secondary metabolism and defence and regulated by light, exhibited a disproportionate change with 41/50 DEGs being down-regulated within 8 h of ethylene treatment. These included key pathway genes like phenylalanine ammonia lyase, chalcone synthases, chalcone isomerases, caffeoyl CoA O-methyl transferases etc. (Table S3). The cytochrome P450 oxidase family which regulates the phenylpropanoid, alkaloid and terpenoid pathways was also differentially affected with 44 DEGs (20 up and 24 down-regulated) (Fig. 4a; Table S3).

Wall hydrolysis is an important component that enables separation of the abscising organ while cell wall reinforcement is needed to protect the tissue exposed after organ separation. In keeping with the complexity of the cell wall, 55 DEGs involved in biosynthesis and modification of cell wall components were differentially regulated. These included genes encoding xyloglucan endotransglucosylase/hydrolases (XTHs), pectinesterases, expansins and pectate lyase etc. Almost 65% of these were prominently down-regulated upon abscission (Fig. 4b; Table S3).

Quite strikingly, a large group of genes (245/1475), encoding transporters for ions (33), amino acids (25), water/aquaporins (11), sugar/sugar phosphates (28), ATP-binding cassette (ABC) transporters (22), heavy metals (13), drug transporters (20) and others (61) were differentially expressed during abscission (Fig. 4c). Most of the sugar, ion, metal and ABC transporter genes were up-regulated in 8 h ethylene-treated AZ, while other transporters related to water, phosphate, amino acid, mitochondrial electron transport, nucleotide and vesicle-mediated membrane transport were down-regulated (Fig. 4c; Table S3).

Another rather surprising observation was how abscission affected the light-regulated, photosynthesis-related genes involved in light reactions in plastids. A large majority of these (49/56) encoding components of photosystems I and II, chlorophyll ab-binding proteins, oxygen evolution, electron transport and oxido-reductases were strongly down-regulated while a chlorophyllase was up-regulated suggesting suppression of several plastid activities. This affected carbohydrate metabolism with 42/70 DEGs involved in glycolysis, gluconeogenesis, TCA cycle, PPP pathway and starch degradation being down-regulated (Table S3).

### Validation of differentially regulated genes in ethylene-treated petal abscission zones

**Figure 5:**
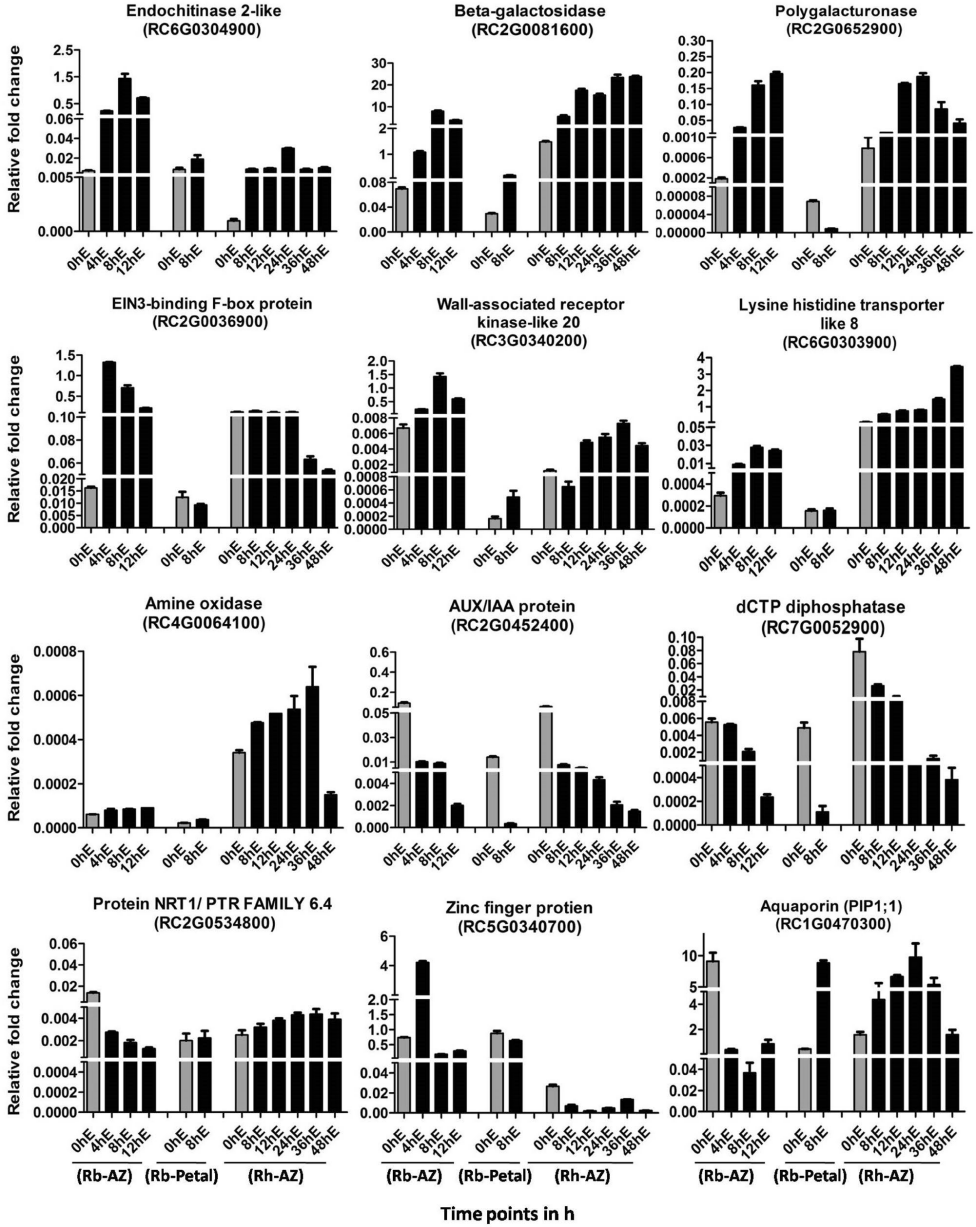
Comparative real time PCR validation of selected DEGs obtained from the transcriptome comparisons. Expression was studied in AZs at 0 h (untreated), 4, 8 and 12 h after 0.5 µl L^−1^ ethylene treatment for *R. bourboniana* and additionally at 24, 36 and 48 h for *R. hybrida*. For petal, expression was studied at 0 h and 8 h after ethylene treatment. The real time expression data was normalized using *ACTIN* as reference*.* Error bars represent ± SE of three biological replicates. Expression values were analyzed by one-way ANOVA and compared using Duncan’s Multiple Range Test (DMRT) for AZ expression analysis. Values on the bar carrying different letters are significantly different. Paired *t* test was applied to analyze expression values in petal tissue. Error bars represent SE, *indicates *P* < 0.05, **indicates *P* < 0.01, ***indicates *P* < 0.001.

We next performed a qRT-PCR validation of expression using 12 randomly selected DEGs from the above categories that were differentially regulated (7 up and 5 down) in the 8 h ethylene-treated petal AZ (Table S5). A more detailed time-course study was performed by including samples at 0, 4, 8 and 12 h after ethylene treatment for *R. bourboniana* and additionally at 24, 36 and 48 h for *R. hybrida* and 0 and 8 h for ethylene-treated petals. In agreement with the transcriptome data, the expression of genes encoding a putative endochitinase 2-like protein (RC6G0304900), beta-galactosidase (RC2G0081600), a polygalacturonase (RC2G0652900), a putative EIN3-binding F-box protein (RC2G0036900), a lysine-histidine transporter-like 8 protein (RC6G0303900), a wall-associated receptor kinase (RC3G0340200) and a putative amine oxidase (RC4G0064100) was induced by ethylene in *R. bourboniana* AZ as seen in the transcriptome and increased from 0 to 12 h post-ethylene treatment during the abscission time-course (Fig. 5). The fold change in real time PCR validation was usually higher than that seen in the transcriptome except for the amine oxidase gene (Table S5). For most, expression also increased in *R. hybrida* AZ but at a later stage and to a lesser extent compared to *R. bourboniana*. In contrast, the transcript abundance of genes encoding a putative AUX/IAA protein (RC2G0452400), a dCTP diphosphatase (RC7G0052900), a putative NRT1/PTR FAMILY 6.4 protein (RC2G0534800) and a putative aquaporin (RC1G0470300) decreased strongly in 8 h ethylene-treated petal AZs of *R. bourboniana* suggesting suppression of these genes by ethylene. While this decrease was also seen in *R. hybrida* for the first two genes, expression of the aquaporin-like gene increased considerably in *R. hybrida* petal AZ and *R. bourboniana* petals while that of the NRT1/PTR FAMILY 6.4 gene increased slightly. The expression of another down-regulated zinc finger protein gene was unusual in that it showed a decline in expression in 8 h ethylene-treated petal AZ, as seen in the transcriptome, but this was preceded by an ethylene-induced increase of ~ fivefold within 4 h.

### Comparative analysis between ethylene-treated *R. bourboniana* petal AZ versus petal and between petal AZs of *R. bourboniana* versus *R. hybrida* for identification of putative AZ-specific genes

**Figure 6:**
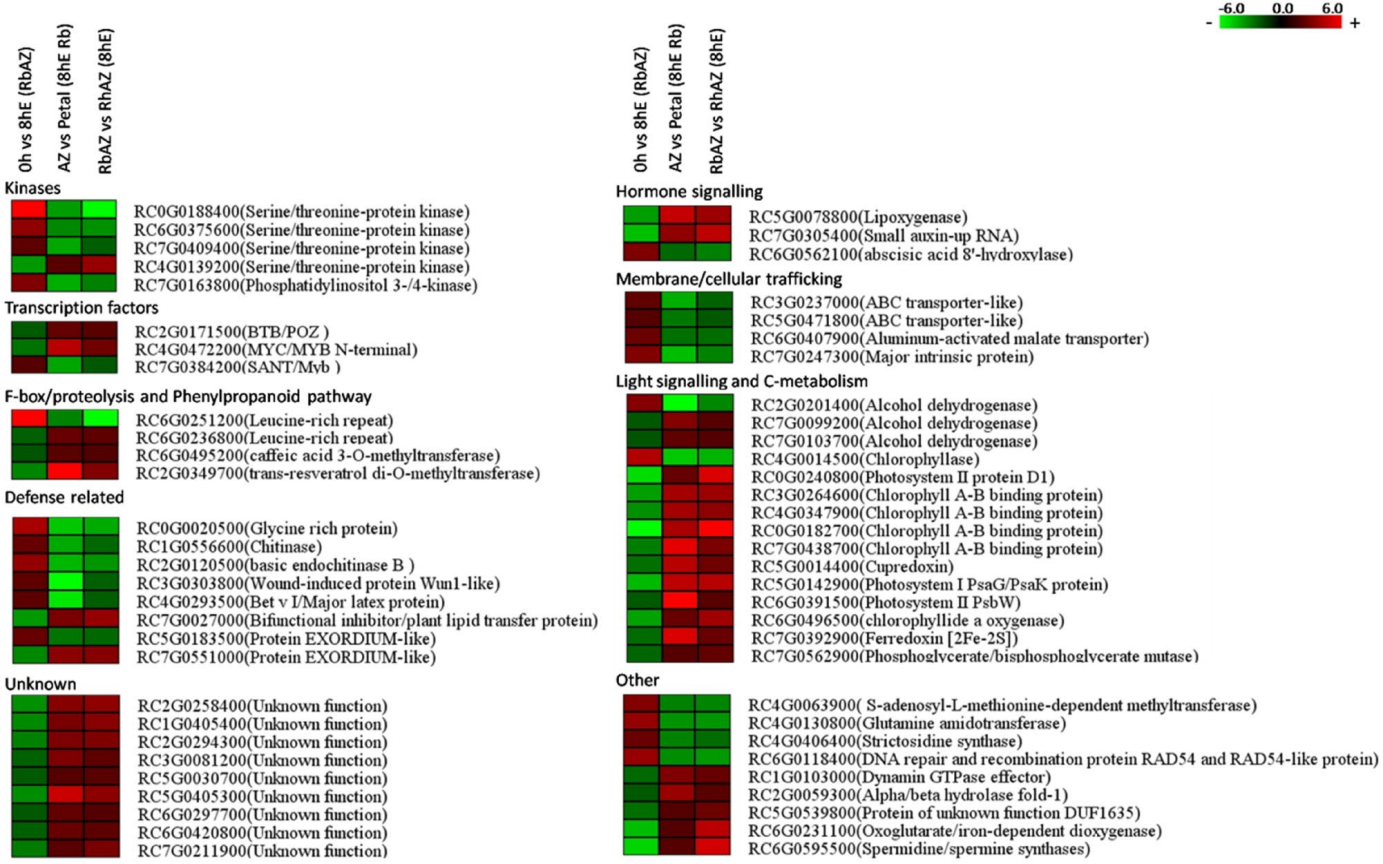
Heat map showing the expression of putative abscission related genes in the comparison of ethylene-treated petal AZ and petals of *R. bourboniana* and petal AZ of *R. hybrida*. The 60 DEGs common to the 0 h versus 8 h ethylene-treated petal AZ transcriptomes in *R. bourboniana* show opposite regulation in the comparison between 8 h ethylene-treated petal AZ versus 8 h ethylene-treated petals of *R. bourboniana* and 8 h ethylene treated petal AZ of *R. hybrida.* Analysis was performed at [log_2_|FC|≤ − 2 and ≥ 2)] and Q-value ≤ 0.05.

The comparison between 0 and 8 h ethylene-treated petal AZs identified genes that are differentially regulated by ethylene during abscission. Since some of these may represent general ethylene-responsive genes, not necessarily associated with abscission, we next performed two additional comparisons. One compared the 8 h ethylene-treated petal AZ with 8 h ethylene-treated petals of *R. bourboniana* to identify genes that were differentially regulated in AZ but not in petals upon ethylene treatment (Table S7). This comparison would allow identification of genes that are abscission-regulated. A second comparison was between 8 h ethylene-treated petal AZ of the ethylene-sensitive *R. bourboniana* with 8 h ethylene-treated petal AZ of the less-sensitive *R. hybrida*. This was to identify genes differentially regulated only in the AZ of *R. bourboniana* (where abscission progresses to the mid-point by 8 h), but not in *R. hybrida* where abscission takes ~ 50 h (Table S8). Of the 6288 DEGs identified in the first comparison with 8 h ethylene-treated *R. bourboniana* petals, 845 genes (384 up, 461 down) were also identified as DEGs in the AZ transcriptome of *R. bourboniana* at log_2_FC <  − 1, > 1. Of these, 181 DEGs (76 up, 105 down) had a log_2_-fold change (|FC|≤ − 2 and ≥  2) (Table S7). These represented DEGs that were specifically regulated by ethylene at 8 h in AZ (Fig. S4). Similarly, of the 5365 DEGs identified in the second comparison with 8 h ethylene-treated *R. hybrida* petal AZ, 790 (270 up, 520 down) were present as DEGs in the 0 versus 8 h ethylene-treated petal AZ transcriptome of *R. bourboniana* at log_2_FC <  − 1, > 1. Of these, 149 DEGs (46 up, 103 down) had a log_2_-fold change (|FC|≤ − 2 and ≥  2) (Table S8). These represented genes that were specifically regulated by ethylene at 8 h in the AZ (Fig. S5). Through further analysis, we identified those genes that were uniquely expressed in AZ of *R. bourboniana* but reciprocally regulated in the petal and *R. hybrida* AZ samples. A total of 255 DEGs were identified at log_2_ [|FC|≤ − 1 and ≥ 1] of which 108 were up-regulated in *R. bourboniana* but down-regulated in the other two samples and 147 DEGs that were down-regulated in *R. bourboniana* but up-regulated in the other two (Table S9). Of these 255, that possibly guide the abscission process, 58 (~ 23%) encoded kinases/TFs and F-box proteins suggesting transcriptional and post-transcriptional regulation as the most important form of regulation during abscission. The MYB/MYC/ERF and Zn finger proteins were the major TFs representing abscission-regulated DEGs. Of these 255 DEGs, 60 showed strong differential regulation even at log_2_ [|FC|≤ − 2 and ≥ 2]. The majority of these (37) were down-regulated while about a third (23) were up-regulated in *R. bourboniana*. These included six protein kinases, three TFs, one F-box protein, four transporters, three hormone pathway components, ten defence/phenylpropanoid pathway/wall separation components, nine encoding different enzymes and nine with unknown functions. Within the above groups, four of the kinase genes, six defence-related genes and all the four transporter genes were up-regulated. Strikingly, a substantial fraction with 15 members (25% of the total) encoded light reaction and photosystem components (Fig. 6, Table S9). Of these, 13 were down-regulated in the AZ while one chlorophyllase was up-regulated (Table S9). These DEGs represented genes that were specifically regulated in *R. bourboniana* petal AZ in response to ethylene and abscission cues.

## Discussion

Plant organ abscission is an important developmental process that regulates reproductive success and productivity^1–4^. Understanding its molecular basis has the potential to increase the economic value of several ornamentals by preventing premature flower/petal abscission. In rose, the fragrant variety is highly sensitive to ethylene and loses petals within 1–2 days post-pollination, decreasing its commercial value. In this study, high-throughput sequencing permitted us to understand the changes occurring within the small specialized AZ tissue that determines organ abscission but has only been studied occasionally^30,49,50,52–54,58,66,67^ due to the relative difficulty in its study.

The *R. bourboniana* transcriptome was assembled using the sequenced *R. chinensis* genome as a reference^68^. A total of 32,961 genes out of 43,301 were expressed in the AZ. Of these, 3700 genes (representing ~ 8.5% of the total) were differentially expressed at log_2_FC ≥ 1, ≤  − 1 (*P* value < 0.05) of which almost 60% (2182 genes) showed a reduction in transcription. This is a much higher number than a previous study where AZ samples were collected at different stages of bud development of *R. chinensis*^30^ in absence of ethylene treatment. Our study confirms that ethylene accelerates abscission in fragrant roses^11,25,61^ by causing a major change in gene expression in the AZ. The expression of reference genes, identified previously for rose^64,65^, did not change in AZs even upon ethylene treatment, indicating their utility as reference for abscission studies (Fig. S2).

The major functional categories of the DEGs included regulatory genes (~ 11% DEGs) encoding transcription factors (284/2220), kinases (203/1849), phosphatases (86/503) and F-box/proteolysis components (218/2335) while other categories included hormone signalling (111)**,** transporters (245) and, to a lesser extent, defense and stress response pathways. Surprisingly, light harvesting components (56/338) and the light-regulated phenylpropanoid pathway were strongly and disproportionately suppressed (Figs. 2, 3, 4, Tables S3, S4). All these groups have been identified previously as differentially regulated during abscission in other plants including rose^12,49,52–54,56,58^ although the relative proportion within each group varies.

One of the major findings of this study has been the rapid transcriptional activation of the ethylene pathway and suppression of auxin and JA pathways within 8 h of ethylene treatment. Ethylene accelerates the abscission process^5,14,69^. Hence, a rapid induction of genes encoding components of ethylene perception and signalling in the present transcriptome (Fig. 3) was not surprising. Our own detailed analysis of the ethylene pathway^25^ confirmed the up-regulation of ethylene biosynthesis and signalling in *R. bourboniana* during abscission but its suppression in *R. hybrida*. Unlike ethylene, endogenous auxin prevents abscission by reducing AZ sensitivity to ethylene^29,70^. A decrease in auxin response in the AZ allows endogenous ethylene to hasten abscission in various organs^71–73^. Thus, down-regulation of most genes of the auxin pathway suggests that it may suppress petal abscission^25^ in rose through similar mechanisms (Table S4; Fig. 3).

Besides ethylene and auxin, JA affects abscission of fruits, leaves and flowers, independently from ethylene^74–77^. JA is known to promote senescence as well as abscission^78–81^*.* The JA-defective *aos* and *coi1* mutants of Arabidopsis, show considerably delayed abscission indicating that JA promotes abscission^34^. It also induces anther dehiscence which, like abscission, requires cell separation^82,83^. In contrast to these studies, a majority of the JA pathway components, from biosynthesis to response, were down-regulated during ethylene-induced abscission in rose. These included JA biosynthesis genes such as *PLA*, *LOX*, *AOC, OPR, JMT* and the JA-responsive MYC homologue that activates JA-responsive genes^84,85^. Several genes encoding JAZ proteins (negative regulators of JA signalling that are transcriptionally up-regulated during JA signaling) were also down-regulated. The results suggested that JA may suppress rose petal abscission, unlike in Arabidopsis. In this context, the strong down-regulation of the light pathway genes of the photosynthetic machinery (discussed later) is interesting. The initial steps in JA biosynthesis occur in the plastid and the down-regulation of many chloroplastic genes may disrupt plastid functioning and may, in some way, be related to the down-regulation of the JA pathway. Interestingly, the connection between JA and light signalling goes further with many aspects of JA responses requiring functional phytochromes and *vice versa*^86^. Conclusions about the nature of the involvement of hormones like GA, ABA, cytokinin and SA were difficult, since genes involved in their biosynthesis as well as catabolism were differentially regulated (Fig. S3).

The activation of abscission-specific genes that drive abscission requires the action of TFs and post-translational regulators encoding kinases, phosphatases, receptors and various F-box proteins. Indeed, TFs of different families like ZF, MYB, MYC, ERF, NAC, HB and WRKY accounted for ~ 5% (284) of the total AZ DEGs highlighting their importance in regulating rose petal abscission (Fig. 2a). Studies of other abscission-related transcriptomes^30,51,54,66^ have also revealed their involvement although only a few members have been characterized to date for a detailed role in abscission^42,45,48,49,87^. Some members of the ERF and MYB/MYC families regulate the ethylene and JA pathways. Interestingly, the ERF family (8 up, 7 down) showed a disproportionately larger number of up-regulated members possibly reflecting the greater involvement of ethylene in abscission while the BTB-POZ family (2 up, 9 down) was more prominently down-regulated as a group suggesting an abscission-inhibitory role. Although very few MADS genes were identified as differentially-regulated, these are important players in determining the AZ development^38,39,48^. Two genes RC4G0415400 and RC7G0528400 showing similarity to the Arabidopsis AGL8 and AGL24 were significantly down-regulated at Log_2_FC <  − 1  (Table S3). *AGL8*, and its tomato homologue *FUL2*, are known to be involved in seed shattering and abscission, respectively, with *FUL2* expression suppressing style abscission in tomato^88,89^. These genes may perform a similar function in rose petal abscission. *AGL24* is the homologue of tomato *JOINTLESS*, a key abscission regulator required for AZ development^48^. Four other MADS genes were significantly down-regulated but with a lesser change. The down-regulation of all these genes in rose probably reflects that these are not needed after formation of the AZ or may inhibit abscission progression.

Protein kinases and phosphatases, with 203 and 86 DEGs respectively (5% of the total DEGs), formed another important group regulating rose petal abscission. Post-translational modifications by these regulate the activity of several enzymes and proteins during developmental processes including abscission^90^. In *Arabidopsis*, receptor-like kinases such as HAESA and HAESA-LIKE2 promote abscission while other RLKs like SOMATIC EMBRYOGENESIS RECEPTOR-LIKE KINASE (SERK) positively regulate floral organ abscission in combination with HAE/HSL2^91^. Two other RLKs—SUPPRESSOR OF BIR1 (SOBIR1/EVERSHED) and CASTAWAY control the extent of cell separation during organ shedding^41,92^. These RLKs, along with MAP kinases, are differentially regulated during the cell separation phase of organ abscission in Arabidopsis^36,37,41,49,93,94^ and in citrus leaf AZ^95^. HSL2 also interacts with another RLK, RLK7, in Arabidopsis^96^. Homologues of *HSL1* (RC7G0442700) and *RLK7* (RC7G0493400) and *MPK*s were differentially regulated in the rose AZ transcriptome. Their presence in the core set of 255 rose petal AZ-specific genes is a strong indication of a conserved function in the abscission machinery across plants. In addition, a disproportionately high number of histidine kinases (12/15) was up-regulated along with a simultaneous down-regulation of histidine phosphatases (4/5) indicating a specific role for this class of proteins in abscission. Histidine kinases are important players of two-component relay systems such as cytokinin signalling as well as ethylene signalling^97^. The reciprocal regulation of the HK/phosphatase family indicates that targets of these proteins may be specific abscission determinants. Indeed, three of the HKs were identified as *ETR3, ETR7* and *ETR8* that are up-regulated by ethylene^25^ while one was identified as a homologue of *AHK4*, the Arabidopsis cytokinin receptor. Surprisingly, this group also contained the tomato orthologues of *PHYA* and *PHYB*, both of which were up-regulated, while the orthologue of *PHYE* was down-regulated. The up-regulation of phytochromes and the strong down-regulation of light-regulated photosystem and phenylpropanpoid components in abscission (Table S9), may indicate the hitherto less studied involvement of light signalling and phytochromes in abscission. Mutants of phytochromes in Arabidopsis have not been noted to be abscission-defective although a tomato *phyAB1B2* mutant showed early fruit ripening and abscission^98^. A disproportionate reduction (11 down, 1 up) in PP2C type phosphatases was also observed in rose petal AZ transcriptome hinting at a specific role in abscission regulation that will require further studies of their targets.

A considerable number (75) of F-box genes and related proteasomal subunit genes which govern the amount and turnover of key proteins in developmental processes was also differentially regulated. Surprisingly, the majority of F-box DEGs (41/75) were up-regulated indicating that removal of abscission inhibitory proteins by these may be a necessary event for activation of the abscission process.

It was interesting to note an over-representation of genes involved in potential transport activity in 8 h ethylene-treated petal AZ (Fig. 4c; Table S3). Transporters selectively govern the access of molecules in or out of the cells, thereby controlling the overall distribution of substances at their site of action or they may enable nutrient transport from the abscising organ to the parent organ prior to organ separation. Although the role of transporters in abscission is not clear, the fact that more than 200 genes encoding amino acid transporters, ABC transporters, ion transporters and drug transporters were differentially regulated during petal abscission in rose is indication of the importance of the transport of various compounds to the abscission process. ABC transporters are involved in membrane trafficking of a variety of substrates including lipids, drugs, sterols and other metabolic products^99^ and hormones^100^. At least 13 putative ABC lipid transporter genes have been reported to differentially express in the citrus fruit calyx AZ upon ethylene treatment^101^ whereas six ABC transporter genes were expressed during leaf abscission in sugarcane^58^. Differential expression of genes involved in transport of sugars, nutrients, metals, nitrate, lipids etc. has also been observed during abscission in olive^56,102^ and citrus^101^ while expression of sugar transporter genes was observed in calyx and pedicel AZ transcriptome profiling of citrus^101^ pyrus^66^ and tomato^52^. Collectively these results suggest that transporters may play a major role in membrane trafficking during abscission across plants.

One of the most prominent sets of DEGs in AZ was associated with the plastid photosystem components. Almost 40/50 DEGs were strongly down-regulated in the abscission transcriptome thus displaying a considerable deviation from the 1:1.5 ratio of up:down genes in the AZ. Although the few cell layer thick AZ would not strike one as a major site for regulation of photosystem-related genes, decrease in expression of photosynthetic genes does appear to be a component of changes associated with onset of fruit abscission^54,103,104^ as well as other AZ transcriptome profiling studies^51,54,66^. In rose, these genes accounted for 25% of the 60 most strongly regulated abscission-specific genes obtained from further comparisons with ethylene-treated *R. bourboniana* petals and *R. hybrida* petal AZ*.* The strong ethylene-induced suppression of the plastidial photosynthetic machinery genes functioning in photosystem organization, light-harvesting, chlorophyll binding, electron transport and carbon fixation in rose petal AZ (Table S3) appears in some way to be necessary for the abscission process to begin or progress. Chloroplasts are the major source for generation of reactive oxygen species (ROS) and a disruption in chloroplast photosynthetic machinery increases ROS and activates senescence^105^. The decrease in expression of various photosystem components during petal abscission in rose may similarly disrupt the photosynthetic electron transport machinery leading to increased ROS generation and abscission although this will require more detailed studies. Another major component preferentially expressed in 8 h ethylene-treated petal AZ of *R. bourboniana* is associated with cell death and disassembly of proteins, nucleic acids and cell wall components. Disassembly of cellular structures have been noted in the context of abscission and observed in previous studies of abscission^102,106–108^ including our own^59,63^ where genes encoding cysteine proteases and components of PCD and autophagy were up-regulated. A large number of cell wall remodelling genes encoding XTHs, expansins, cellulose synthases, pectate lyases and arabinogalactan proteins probably contribute to cell separation and fortification of the wall after abscission^1,11,12,49,50,55,60–62,109–114^ and was also observed in rose. Since organ separation exposes the nascent tissue to pathogens, protection of this tissue is necessary and is associated with expression of a large number of defence-related genes^12,13,49,50,115–118^. Genes encoding PR-proteins and those associated with the reactive oxygen species (ROS) pathway, and oxidative burst such as peroxidases were also up-regulated in rose (Table S3).

Further analysis to obtain genes specific to *R. bourboniana* AZ was carried out with comparisons with ethylene-treated *R. bourboniana* petals to exclude genes represented in petals (with which the petal AZ is closely attached) and those represented in ethylene-treated petal AZ of *R. hybrida* (which shows reduced ethylene sensitivity and delayed abscission). The analysis revealed a total of 845 and 790 DEGs respectively (at log_2_FC ≥ 1, ≤  − 1, *P* value < 0.05; Tables S7, S8) within which 255 DEGs were identified as being part of a core set of genes that were regulated by ethylene only in *R. bourboniana* petal AZ but not in petals and *R. hybrida* AZ*.* Strikingly, 58/255 genes (~ 23%) belonged to the regulatory category consisting of TFs/kinases/phosphatases/F-box proteins. This is much higher than the proportion (11%) observed in comparison between 0 and 8 h ethylene-treated petal AZs and suggests a specific requirement for this group in abscission (Table S9). The TF subset accounting for 10% of these genes included members of the AP2-ERF/MYB/MYC/Zn finger families. About 60/255 DEGs were uniquely regulated in AZs at log_2_FC ≥ 2, ≤  − 2 (*P* value < 0.05). The majority of the kinases, defence-related and the transporter genes in this group were up-regulated (Table S9) while 13 of the 15 photosystem-related genes were strongly down-regulated. The down-regulation of these plastid-associated genes along with the light-regulated phenylpropanoid pathway indicates that light-associated components may have a strong negative effect on progression of abscission and is currently under study. A comparison of this core set of 255 genes with Arabidopsis reveals that at least 58 have homologues in Arabidopsis that show abscission-related expression in Arabidopsis siliques and floral parts as shown in Genevisible (https://genevisible.com/search) and other studies^49,56,89,119–124^ (Table S9). Interestingly, these include two TFs, WIN1 and MYB94 both involved in wax biosynthesis^120,123^, MYB117, WRKY33^121^ (a PAMP and SA responsive TF), seven receptor kinases^49,119^, eight transporters and several genes encoding different enzymes. The functions of all these genes in the context of abscission are not clear and will require future knock-out and over-expression studies. Nevertheless, the studies suggest that a large fraction of the abscission machinery representing different functional groups is conserved across families as diverse as rose and Arabidopsis, despite tissue differences.

In conclusion, ethylene-induced rose petal abscission is associated with large scale gene expression changes distinct from those in the petal. It appears to be guided by changes in responses of the ethylene, auxin and JA pathways and related ERFs/MYB/MYCs that may affect sensitivities to these hormones. The expression of phytochromes and the prominent suppression of light-associated photosystem and phenylpropanoid components suggest a close interaction of light signalling and plastid with abscission. Expression of regulatory genes like TFs, kinases/phosphatases/F-box genes that include homologues of MADS genes *AGL8*, *AGL24* and RLKs like *HSL1* and *RLK7* besides transporters and defense pathways suggest some conservation with Arabidopsis with certain components like the JA pathway being regulated in a unique manner in rose.

## Material and methods

### Plant material, treatments and RNA isolation

Flowers of *Rosa bourboniana* (cv Gruss an Teplitz) and *R. hybrida* (Opening Night) were chosen for study. Excised flowers were treated with 0.5 µl/L exogenous ethylene to trigger abscission which is completed in 16–18 h in *R. bourboniana* but 48–50 h in *R. hybrida*. The 8 h time point represents the mid-point of abscission. RNA was isolated from *R. bourboniana* petal AZ samples collected 0, 4, 8 and 12 h after ethylene treatment of flowers and 8 h ethylene-treated whole petals and from petal AZ of 0, 8, 12, 24, 36 and 48 h ethylene-treated *R. hybrida* flowers in three independent biological sets as described^11,60^ using the plant total RNA isolation kit (Sigma). The integrity of RNA was assessed by electrophoresis on a 1.2% agarose gel in 0.5X TBE. RNA samples with RIN (RNA integrity number) value more than 6.0, 260/280 ratios from 1.8 to 1.9 and 260/230 ratios from 2.0 to 2.5 were used further for Illumina sequencing and expression studies.

### Illumina sequencing

The cDNA libraries were generated from mRNA of the above samples. The 0 h and 8 h ethylene-treated petal AZ RNA from *R. bourboniana*, the 8 h ethylene-treated petal AZ RNA from *R. hybrida* and ethylene-treated petal RNA of *R. bourboniana* were used for paired-end sequencing (with 100 bp ends) on an Illumina HiSeq2000 sequencing machine (Scigenome, Kochi, India). About 45–67 million reads were generated per sample. Low quality sequences and adaptor sequences were removed and reads trimmed by NGSQCTOOLKIT (https://www.nipgr.res.in/ngsqctoolkit.html). More than 85% high quality reads with a quality score (Q > 30) value were generated from each library for analysis.

### Assembly and gene expression analysis

The filtered high quality reads were used in reference-guided assembly using *R. chinensis*^68^ genome as reference with TopHat v2.1.1^125^ (https://ccb.jhu.edu/software/tophat/index.shtml) and Bowtie programs^126,127^ as alignment tools and SAM Tools^128^ for processing the alignment file. The aligned reads were counted and estimated for gene expression as FPKM using cufflinks v2.2.1 software^129^. Cuffdiff was used to estimate genes showing significant differential expression using the Cufflinks package^129^ (http:/ial/cole-trapnell-lab.github.io/cufflinks/manual/).

### Functional annotation

To identify the putative functions, assembled transcripts were annotated using GO^130^, KEGG^131,132^ (https://www.genome.jp/kegg/) and InterPro (ftp://ftp.bioinfo.wsu.edu/species/Rosa_chinensis/Rchinensisgenome.v1.0/functional/) and integrated with our result using in-house custom scripts in R programming language. A heat map was generated for the differentially regulated contigs using MeV version 4.9 (https://www.tm4.org/mev.html). DEGs with Log_2_|FC|(≤ − 1 and ≥ 1) were grouped under various categories for functional cataloguing (Figs. 2, 3, 4). For analysis of common genes in all three comparisons, DEGs with Log_2_|FC|(≤ − 2 and ≥ 2) were used (Fig. 6).

### Validation of mRNA-seq data using qRT-PCR

Real time PCR was performed to quantify the expression of selected DEGs (Table S5) obtained from Illumina sequencing. The cDNAs, used as template, were generated from mRNA using the REVERTAID MMLV reverse transcriptase (Fermentas). Primers (Table S6) were designed using the primer designing tool at IDT (https://eu.idtdna.com/site) to amplify an amplicon of 80–150 nucleotides with Tm around 60 °C. Reactions were run in triplicates (technical and biological) for each sample using Power-Up SYBR Green on an ABI StepOnePlus real time PCR machine (Applied Biosystems Inc, USA) and the data analyzed was the mean of biological triplicates. The reaction was set up in 20 µl as follows: 1 µl of cDNA, 10 µl SYBR Green Dye master mix (2X), 5 pmol each of forward and reverse primers and water up to 20 µl. The general steps performed during real-time PCR experiment were as follows: step 1, 50 °C, 2 min, step 2, 95 °C, 10 min, step 3 (95 °C 15 s, 60 °C 1 min) × 40 cycles. The specificity of the amplicon was analyzed by a melt curve analysis. The relative mRNA level of the gene in different RNA samples was normalized with respect to *ACTIN* as the internal control gene^60^ and analyzed by 2^−∆CT^ method^133^.

## Supplementary information

Supplementary Legends

Supplementary Information 2

Supplementary Information 3

Supplementary Information 4

Supplementary Information 5

Supplementary Information 6

Supplementary Information 7

Supplementary Information 8

Supplementary Information 9

## Data Availability

The sequence data has been submitted to NCBI under the SRA accession: PRJNA594099 (Temporary Submission ID: SUB6655877), Release date: 2021-01-04.

## References

[1] Roberts JA (2002). Abscission, dehiscence, and other cell separation processes. Annu. Rev. Plant Biol..

[2] Addicott FT (1982). Abscission.

[3] Estornell LH (2013). Elucidating mechanisms underlying organ abscission. Plant Sci..

[4] Nakano T, Ito Y (2013). Molecular mechanisms controlling plant organ abscission. Plant Biotechnol..

[5] Meir S (2019). Re-evaluation of the ethylene-dependent and-independent pathways in the regulation of floral and organ abscission. J. Exp. Bot..

[6] Roberts JA (1984). Ethylene-promoted tomato flower abscission and the possible involvement of an inhibitor. Planta.

[7] del Campillo E, Lewis LN (1992). Identification and kinetics of accumulation of proteins induced by ethylene in bean abscission zones. Plant Physiol..

[8] Cameron AC, Reid MS (2001). 1-MCP blocks ethylene-induced petal abscission of Pelargonium peltatum but the effect is transient. Postharvest Biol. Technol..

[9] Cin VD (2005). Ethylene biosynthesis and perception in apple fruitlet abscission (Malus domestica L. Borck). J. Exp. Bot..

[10] Macnish AJ (2005). Anatomy of ethylene-induced floral-organ abscission in Chamelaucium uncinatum (Myrtaceae). Aust. J. Bot..

[11] Sane AP (2007). Petal abscission in rose (Rosa bourboniana var Gruss an Teplitz) is associated with the enhanced expression of an alpha expansin gene, RbEXPA1. Plant Sci..

[12] Agustí J (2008). Ethylene-induced differential gene expression during abscission of citrus leaves. J. Exp. Bot..

[13] Bleecker AB, Patterson SE (1997). Last exit: senescence, abscission, and meristem arrest in Arabidopsis. Plant Cell.

[14] Patterson SE, Bleecker AB (2004). Ethylene-dependent and-independent processes associated with floral organ abscission in Arabidopsis. Plant Physiol..

[15] Hall AE, Bleecker AB (2003). Analysis of combinatorial loss-of-function mutants in the Arabidopsis ethylene receptors reveals that the ers1 etr1 double mutant has severe developmental defects that are EIN2 dependent. Plant Cell.

[16] Lanahan MB (1994). The never ripe mutation blocks ethylene perception in tomato. Plant Cell.

[17] Tieman DM (2001). Members of the tomato LeEIL (EIN3-like) gene family are functionally redundant and regulate ethylene responses throughout plant development. Plant J..

[18] Whitelaw CA (2002). Delayed abscission and shorter internodes correlate with a reduction in the ethylene receptor LeETR1 transcript in transgenic tomato. Plant Physiol..

[19] Okabe Y (2011). Tomato TILLING technology: development of a reverse genetics tool for the efficient isolation of mutants from Micro-Tom mutant libraries. Plant Cell Physiol..

[20] Müller R (2000). Characterization of an ethylene receptor family with differential expression in rose (Rosa hybrida L.) flowers. Plant Cell Rep..

[21] Müller R (2000). Stress induced ethylene production, ethylene binding, and the response to the ethylene action inhibitor 1-MCP in miniature roses. Sci. Hortic..

[22] Ruperti B (2001). Characterization and expression of two members of the peach 1-aminocyclopropane-1-carboxylate oxidase gene family. Physiol. Plant.

[23] Rasori A (2003). Functional analysis of peach ACC oxidase promoters in transgenic tomato and in ripening peach fruit. Plant Sci..

[24] Hilt C, Bessis R (2003). Abscission of grapevine fruitlets in relation to ethylene biosynthesis. VITIS-GEILWEILERHOF.

[25] Singh P (2019). Differential and reciprocal regulation of ethylene pathway genes regulates petal abscission in fragrant and non-fragrant roses. Plant Sci..

[26] Jin X (2015). Auxin is a long-range signal that acts independently of ethylene signaling on leaf abscission in Populus. Front. Plant Sci..

[27] Ellis CM (2005). AUXIN RESPONSE FACTOR1 and AUXIN RESPONSE FACTOR2 regulate senescence and floral organ abscission in Arabidopsis thaliana. Development.

[28] Basu MM (2013). The manipulation of auxin in the abscission zone cells of Arabidopsis flowers reveals that indoleacetic acid signaling is a prerequisite for organ shedding. Plant Physiol..

[29] Meir S (2015). Role of auxin depletion in abscission control. Stewart Postharvest Rev..

[30] Gao Y (2016). Transcriptome profiling of petal abscission zone and functional analysis of an Aux/IAA family gene RhIAA16 involved in petal shedding in rose. Front. Plant Sci..

[31] Porter NG, Van Steveninck RFM (1966). An abscission-promoting factor in *Lupinus luteus* (L.). Life Sci..

[32] Hartmann HT, Heslop A, Whisler J (1968). Chemical induction of fruit abscission in olives. Calif. Agric..

[33] Zucconi F (1969). Promotion of fruit abscission with abscisic acid. Bioscience.

[34] Kim J (2013). New clothes for the jasmonic acid receptor COI1: delayed abscission, meristem arrest and apical dominance. PLoS ONE.

[35] Butenko MA (2003). Inflorescence deficient in abscission controls floral organ abscission in Arabidopsis and identifies a novel family of putative ligands in plants. Plant Cell.

[36] Jinn TL (2000). HAESA, an Arabidopsis leucine-rich repeat receptor kinase, controls floral organ abscission. Genes Dev..

[37] Cho SK (2008). Regulation of floral organ abscission in *Arabidopsis thaliana*. Proc. Natl. Acad. Sci..

[38] Fernandez DE (2000). The embryo MADS domain factor AGL15 acts postembryonically: inhibition of perianth senescence and abscission via constitutive expression. Plant Cell.

[39] Adamczyk BJ (2007). The MADS domain factors AGL15 and AGL18 act redundantly as repressors of the floral transition in Arabidopsis. Plant J..

[40] Liljegren SJ (2009). Regulation of membrane trafficking and organ separation by the NEVERSHED ARF-GAP protein. Development.

[41] Leslie ME (2010). The EVERSHED receptor-like kinase modulates floral organ shedding in Arabidopsis. Development.

[42] Chen MK (2011). The MADS box gene, FOREVER YOUNG FLOWER, acts as a repressor controlling floral organ senescence and abscission in Arabidopsis. Plant J..

[43] Li C (2006). Genetic analysis of rice domestication syndrome with the wild annual species, Oryza nivara. New Phytol..

[44] Konishi S (2006). An SNP caused loss of seed shattering during rice domestication. Science.

[45] Zhou Y (2012). Genetic control of seed shattering in rice by the APETALA2 transcription factor SHATTERING ABORTION1. Plant Cell.

[46] Yoon J (2014). The BEL 1-type homeobox gene SH 5 induces seed shattering by enhancing abscission-zone development and inhibiting lignin biosynthesis. Plant J..

[47] Butler L (1936). Inherited characters in the tomato II. Jointless pedicel. J. Hered..

[48] Mao L (2000). JOINTLESS is a MADS-box gene controlling tomato flower abscission zone development. Nature.

[49] Cai S, Lashbrook CC (2008). Stamen abscission zone transcriptome profiling reveals new candidates for abscission control: enhanced retention of floral organs in transgenic plants overexpressing Arabidopsis ZINC FINGER PROTEIN2. Plant Physiol..

[50] Meir S (2010). Microarray analysis of the abscission-related transcriptome in the tomato flower abscission zone in response to auxin depletion. Plant Physiol..

[51] Wang X (2013). Transcriptome analysis of tomato flower pedicel tissues reveals abscission zone-specific modulation of key meristem activity genes. PLoS ONE.

[52] Nakano T (2013). Expression profiling of tomato pre-abscission pedicels provides insights into abscission zone properties including competence to respond to abscission signals. BMC Plant Biol..

[53] Sundaresan S (2016). De novo transcriptome sequencing and development of abscission zone-specific microarray as a new molecular tool for analysis of tomato organ abscission. Front. Plant Sci..

[54] Zhu H (2011). Transcriptomics of shading-induced and NAA-induced abscission in apple (Malus domestica) reveals a shared pathway involving reduced photosynthesis, alterations in carbohydrate transport and signaling and hormone crosstalk. BMC Plant Biol..

[55] Corbacho J (2013). Transcriptomic events involved in melon mature-fruit abscission comprise the sequential induction of cell-wall degrading genes coupled to a stimulation of endo and exocytosis. PLoS ONE.

[56] Gil-Amado JA, Gomez-Jimenez MC (2013). Transcriptome analysis of mature fruit abscission control in olive. Plant Cell Physiol..

[57] Li C (2013). De novo assembly and characterization of fruit transcriptome in Litchi chinensis Sonn and analysis of differentially regulated genes in fruit in response to shading. BMC Genom..

[58] Li M (2016). De novo analysis of transcriptome reveals genes associated with leaf abscission in sugarcane (Saccharum officinarum L.). BMC Genom..

[59] Tripathi SK (2009). Transcriptional activation of a 37 kDa ethylene responsive cysteine protease gene, RbCP1, is associated with protein degradation during petal abscission in rose. J. Exp. Bot..

[60] Singh AP (2011). Transcriptional activation of a pectate lyase gene, RbPel1, during petal abscission in rose. Postharvest Biol. Technol..

[61] Singh AP (2011). Petal abscission in rose is associated with the differential expression of two ethylene-responsive xyloglucan endotransglucosylase/hydrolase genes, RbXTH1 and RbXTH2. J. Exp. Bot..

[62] Singh AP (2013). Differential expression of several xyloglucan endotransglucosylase/hydrolase genes regulates flower opening and petal abscission in roses. AoB Plants.

[63] Singh P (2019). Petal abscission in roses is associated with the activation of a truncated version of the animal PDCD4 homologue, RbPCD1. Plant Sci..

[64] Klie M, Debener T (2011). Identification of superior reference genes for data normalisation of expression studies via quantitative PCR in hybrid roses (Rosa hybrida). BMC Res. Notes.

[65] Guénin S (2009). Normalization of qRT-PCR data: the necessity of adopting a systematic, experimental conditions-specific, validation of references. J. Exp. Bot..

[66] Qi X (2013). Identifying the candidate genes involved in the calyx abscission process of'Kuerlexiangli’(Pyrus sinkiangensis Yu) by digital transcript abundance measurements. BMC Genom..

[67] Kim J (2016). Transcriptome analysis of soybean leaf abscission identifies transcriptional regulators of organ polarity and cell fate. Front. Plant Sci..

[68] Saint-Oyant LH (2018). A high-quality genome sequence of *Rosa chinensis* to elucidate ornamental traits. Nat. Plants.

[69] Jackson MB, Osborne DJ (1970). Ethylene, the natural regulator of leaf abscission. Nature.

[70] Blanusa T (2005). The regulation of sweet cherry fruit abscission by polar auxin transport. Plant Growth Regul..

[71] Morgan PW, Durham JI (1972). Abscission: potentiating action of auxin transport inhibitors. Plant Physiol..

[72] Wien, H. C. et al. The influence of auxin transport inhibitor placement on stress-induced flower abscission in Capsicum. Progress in Plant Growth Regulation. Springer, Dordrecht 446–452 (1992).

[73] Rungruchkanont K (2007). Endogenous auxin regulates the sensitivity of Dendrobium (cv. Miss Teen) flower pedicel abscission to ethylene. Funct. Plant Biol..

[74] Kubigsteltig I (1999). Structure and regulation of the Arabidopsis thaliana allene oxide synthase gene. Planta.

[75] von Malek B (2002). The Arabidopsis male-sterile mutant dde2-2 is defective in the ALLENE OXIDE SYNTHASE gene encoding one of the key enzymes of the jasmonic acid biosynthesis pathway. Planta.

[76] Hartmond U (2000). Citrus fruit abscission induced by methyl-jasmonate. J. Am. Soc. Hortic. Sci..

[77] Jibran R (2017). Arabidopsis AGAMOUS regulates sepal senescence by driving jasmonate production. Front. Plant Sci..

[78] He Y (2002). Evidence supporting a role of jasmonic acid in Arabidopsis leaf senescence. Plant Physiol..

[79] Schommer C (2008). Control of jasmonate biosynthesis and senescence by miR319 targets. PLoS Biol..

[80] Ueda J (1996). Jasmonates promote abscission in bean petiole expiants: its relationship to the metabolism of cell wall polysaccharides and cellulase activity. J. Plant Growth Regul..

[81] Kim J (2015). To grow old: regulatory role of ethylene and jasmonic acid in senescence. Front. Plant Sci..

[82] Ishiguro S (2001). The DEFECTIVE IN ANTHER DEHISCENCE1 gene encodes a novel phospholipase A1 catalyzing the initial step of jasmonic acid biosynthesis, which synchronizes pollen maturation, anther dehiscence, and flower opening in Arabidopsis. Plant Cell.

[83] Xiao Y (2014). OsJAR1 is required for JA-regulated floret opening and anther dehiscence in rice. Plant Mol. Biol..

[84] De Bruxelles GL, Roberts MR (2001). Signals regulating multiple responses to wounding and herbivores. Crit. Rev. Plant Sci..

[85] Huang H (2017). Jasmonate action in plant growth and development. J. Exp. Bot..

[86] Robson F (2010). Jasmonate and phytochrome A signaling in Arabidopsis wound and shade responses are integrated through JAZ1 stability. Plant Cell.

[87] Norberg M (2005). The BLADE ON PETIOLE genes act redundantly to control the growth and development of lateral organs. Development.

[88] Wang S (2014). Members of the tomato FRUITFULL MADS-box family regulate style abscission and fruit ripening. J. Exp. Bot..

[89] Gu Q (1998). The FRUITFULL MADS-box gene mediates cell differentiation during Arabidopsis fruit development. Development.

[90] Hunter T (1995). Protein kinases and phosphatases: the yin and yang of protein phosphorylation and signaling. Cell.

[91] Meng X (2016). Ligand-induced receptor-like kinase complex regulates floral organ abscission in Arabidopsis. Cell Rep..

[92] Burr CA (2011). CAST AWAY, a membrane-associated receptor-like kinase, inhibits organ abscission in Arabidopsis. Plant Physiol..

[93] Tarutani Y (2004). Molecular characterization of two highly homologous receptor-like kinase genes, RLK902 and RKL1, in Arabidopsis thaliana. Biosci. Biotechnol. Biochem..

[94] Stenvik GE (2008). The EPIP peptide of INFLORESCENCE DEFICIENT IN ABSCISSION is sufficient to induce abscission in Arabidopsis through the receptor-like kinases HAESA and HAESA-LIKE2. Plant Cell.

[95] Agustí J (2009). Comparative transcriptional survey between laser-microdissected cells from laminar abscission zone and petiolar cortical tissue during ethylene-promoted abscission in citrus leaves. BMC Plant Biol..

[96] Olsson, V. et al. The IDA cell separation pathway connects developmental and defense responses. bioRxiv 761346 (2019).

[97] Nongpiur R (2012). Histidine kinases in plants: cross talk between hormone and stress responses. Plant Signal. Behav..

[98] Gupta SK (2014). Complex and shifting interactions of phytochromes regulate fruit development in tomato. Plant Cell Environ..

[99] Terol J (2007). Analysis of 13000 unique Citrus clusters associated with fruit quality, production and salinity tolerance. BMC Genom..

[100] Campbell EJ (2003). Pathogen-responsive expression of a putative ATP-binding cassette transporter gene conferring resistance to the diterpenoid sclareol is regulated by multiple defense signaling pathways in Arabidopsis. Plant Physiol..

[101] Cheng C (2015). Profiling gene expression in citrus fruit calyx abscission zone (AZ-C) treated with ethylene. Mol. Genet. Genom..

[102] Bar-Dror T (2011). Programmed cell death occurs asymmetrically during abscission in tomato. Plant Cell.

[103] Yuan R, Greene DW (2000). Benzyladenine as A chemical thinner for McIntosh'Apples. I. fruit thinning effect 1s and associated relationships with photosynthesis, assimilate translocation, and nonstructural carbohydrates. J. Am. Soc. Hortic. Sci..

[104] Mesejo C (2012). Synthetic auxin 3, 5, 6-TPA provokes citrus clementina (Hor0.t ex Tan) fruitlet abscission by reducing photosynthate availability. J. Plant Growth Regul..

[105] Mayta ML (2018). Expression of a plastid-targeted flavodoxin decreases chloroplast reactive oxygen species accumulation and delays senescence in aging tobacco leaves. Front. Plant Sci..

[106] Evensen KB (1993). Anatomy of ethylene-induced petal abscission in Pelargonium× hortorum. Ann. Bot..

[107] Pérez-Amador MA (2000). Identification of BFN1, a bifunctional nuclease induced during leaf and stem senescence in Arabidopsis. Plant Physiol..

[108] Lers A (2006). Suppression of LX ribonuclease in tomato results in a delay of leaf senescence and abscission. Plant Physiol..

[109] Kim J (2015). Examination of the abscission-associated transcriptomes for soybean, tomato, and Arabidopsis highlights the conserved biosynthesis of an extensible extracellular matrix and boundary layer. Front. Plant Sci..

[110] Taylor JE, Whitelaw CA (2001). Signals in abscission. New Phytol..

[111] Lashbrook CC (1998). Transgenic analysis of tomato endo-β-1, 4-glucanase gene function. Role of cel1 in floral abscission. Plant J..

[112] del Campillo E, Bennett AB (1996). Pedicel breakstrength and cellulase gene expression during tomato flower abscission. Plant Physiol..

[113] Cho HT, Cosgrove DJ (2000). Altered expression of expansin modulates leaf growth and pedicel abscission in Arabidopsis thaliana. Proc. Natl. Acad. Sci..

[114] Ogawa M (2009). ARABIDOPSIS DEHISCENCE ZONE POLYGALACTURONASE1 (ADPG1), ADPG2, and QUARTET2 are polygalacturonases required for cell separation during reproductive development in Arabidopsis. Plant Cell.

[115] Sakamoto M (2008). Involvement of hydrogen peroxide in leaf abscission signaling, revealed by analysis with an in vitro abscission system in Capsicum plants. Plant J..

[116] Goldental-Cohen S (2017). Ethephon induced oxidative stress in the olive leaf abscission zone enables development of a selective abscission compound. BMC Plant Biol..

[117] Dakora FD, Phillips DA (1996). Diverse functions of isoflavonoids in legumes transcend anti-microbial definitions of phytoalexins. Physiol. Mol. Plant Pathol..

[118] Kliebenstein DJ (2004). Secondary metabolites and plant/environment interactions: a view through Arabidopsis thaliana tinged glasses. Plant, Cell Environ..

[119] Wu Y (2016). Genome-wide expression pattern analyses of the Arabidopsis leucine-rich repeat receptor-like kinases. Mol. Plant.

[120] Lee SB, Suh MC (2015). Cuticular wax biosynthesis is up-regulated by the MYB94 transcription factor in Arabidopsis. Plant Cell Physiol..

[121] Lippok B (2007). Expression of AtWRKY33 encoding a pathogen-or PAMP-responsive WRKY transcription factor is regulated by a composite DNA motif containing W box elements. Mol. Plant Microbe Interact..

[122] Farage-Barhom S (2008). Expression analysis of the BFN1 nuclease gene promoter during senescence, abscission, and programmed cell death-related processes. J. Exp. Bot..

[123] Aharoni A (2004). The SHINE clade of AP2 domain transcription factors activates wax biosynthesis, alters cuticle properties, and confers drought tolerance when overexpressed in Arabidopsis. Plant Cell.

[124] Kim SJ (2007). Expression of cinnamyl alcohol dehydrogenases and their putative homologues during Arabidopsis thaliana growth and development: lessons for database annotations?. Phytochemistry.

[125] Trapnell C (2009). TopHat: discovering splice junctions with RNA-Seq. Bioinformatics.

[126] Langmead B (2009). Ultrafast and memory-efficient alignment of short DNA sequences to the human genome. Genome Biol..

[127] Langmead B, Salzberg SL (2012). Fast gapped-read alignment with Bowtie 2. Nat. Methods.

[128] Li H (2009). The sequence alignment/map format and SAMtools. Bioinformatics.

[129] Trapnell C (2010). Transcript assembly and quantification by RNA-Seq reveals unannotated transcripts and isoform switching during cell differentiation. Nat. Biotechnol..

[130] Du Z (2010). agriGO: a GO analysis toolkit for the agricultural community. Nucleic Acids Res..

[131] Kanehisa M (2016). KEGG as a reference resource for gene and protein annotation. Nucleic Acids Res..

[132] Kanehisa M, Goto S (2000). KEGG: kyoto encyclopedia of genes and genomes. Nucleic Acids Res..

[133] Livak KJ, Schmittgen TD (2001). Analysis of relative gene expression data using real-time quantitative PCR and the 2^−ΔΔCT^ method. Methods.

