# Supplementary material for: Petal abscission in fragrant roses is associated with large scale differential regulation of the abscission zone transcriptome
Source: Sci Rep. 2020 Oct 14;10:17196. doi: 10.1038/s41598-020-74144-3 (PMC7566604; doi:10.1038/s41598-020-74144-3)
Supplement: Supplementary file 1 — Supplementary Legends [file 41598_2020_74144_MOESM1_ESM.docx]

**Supplementary File**

**Supplementary figures and tables**

**Supplementary Figure S1:** FPKM plots showing comparison of biological triplicates.

**Supplementary Figure S2:** Heat map of expression of a few reference genes identified for qPCR.

**Supplementary Figure S3:** Expression profile of abscission-related DEGs of the ABA (a), cytokinin (b), GA (c) and salicylic acid (d) pathways.

**Supplementary Figure S4:** Heat map of the 181 differentially expressed genes in different categories obtained from the comparison between 8 h ethylene-treated petal AZ and 8 h ethylene-treated petals of *R. bourboniana*.

**Supplementary Figure S5:** Heat map of the 149 differentially expressed genes in different categories obtained from the comparison between 8 h ethylene-treated petal AZ of *R. bourboniana* and *R. hybrida*.

**Supplementary Table S1:** Summary of reads generated and mapping on rose transcriptome.

**Supplementary Table S2:** All DEGs from the comparisons between 0 h (ethylene-untreated) vs 8 h ethylene-treated petal AZ from *R. bourboniana*, 8 h ethylene-treated petal AZ vs 8 h ethylene-treated whole petals from *R. bourboniana* and 8 h ethylene-treated petal AZ from *R. bourboniana* vs *R. hybrida*.

**Supplementary Table S3:** List of significant DEGs in 0 h (ethylene-untreated) vs 8 h ethylene-treated petal AZ from *R. bourboniana* of various categories such as transcription factors, kinases, phosphatases, F-box/proteolysis, cell wall modification, defence, light signalling, C-metabolism, Ca^+2^ signalling, cytochrome, phenypropanoid pathway and transporters.

**Supplementary Table S4:** List of significant DEGs of hormone signalling pathways with the relative expression levels in 0 h (ethylene-untreated) vs 8 h ethylene-treated petal AZ from *R. bourboniana*.

**Supplementary Table S5:** List of selected genes chosen for real time PCR validation.

**Supplementary Table S6:** List of genes and primers used for study.

**Supplementary Table S7:** List of common DEGs at (log_2_|FC| ≤−1 and ≥1 and log_2_|FC| ≤−2 and ≥2) and Q-value ≤0.05 obtained from the comparison between 0 h vs 8 h ethylene-treated *R. bourboniana* petal AZ samples with 8 h ethylene-treated *R. bourboniana* petal AZ samples vs 8 h ethylene-treated *R. bourboniana* petals (from table S2).

**Supplementary Table S8:** List of common DEGs at (log_2_|FC| ≤−1 and ≥1 and log_2_|FC| ≤ −2 and ≥2) and Q-value ≤0.05 obtained from the comparison between 0 h vs 8 h ethylene-treated *R. bourboniana* petal AZ samples with 8 h ethylene-treated *R. bourboniana* petal AZ samples vs 8 h ethylene-treated *R. hybrida* petal AZ (from table S2).

**Supplementary Table S9**: List of common DEGs in Table S7 and Table S8 that are up-regulated in *R. bourboniana* petal AZ but down-regulated in *R. bourboniana* petals and *R. hybrida* petal AZ and those that are down-regulated in *R. bourboniana* petal AZ but up-regulated in *R. bourboniana* petals and *R. hybrida* petal AZ at (log_2_|FC| ≤−1 and ≥1) and log_2_|FC| ≤−2 and ≥2) and Q-value ≤0.05. The accession numbers of genes identified in Arabidopsis as AZ-related are also shown.
