# Supplementary material for: Petal abscission in fragrant roses is associated with large scale differential regulation of the abscission zone transcriptome
Source: Sci Rep. 2020 Oct 14;10:17196. doi: 10.1038/s41598-020-74144-3 (PMC7566604; doi:10.1038/s41598-020-74144-3)

**Supplementary File**

**Petal abscission in fragrant roses is associated with large scale differential regulation of the abscission zone transcriptome**

Priya Singh^1,2*^, Neeraj Bharti^3,4^, Amar Pal Singh^1,5^, Siddharth Kaushal Tripathi^1,6^, Saurabh Prakash Pandey^1,2^, Abhishek Singh Chauhan^1,2^, Abhijeet Kulkarni^3^ and Aniruddha P. Sane^1,2*^

^1^Molecular Biology and Biotechnology, CSIR-National Botanical Research Institute (CSIR), Lucknow-226001, India,

^2^Academy of Scientific and Innovative Research (AcSIR), Ghaziabad-201002, India,

^3^Bioinformatics Centre, Savitribai Phule Pune University, Pune - 411007, India,

^4^High Performance Computing-Medical and Bioinformatics Applications Group, Centre for Development of Advanced Computing, Pune-411008, India,

^5^Current address: National Institute for Plant Genome Research, New Delhi-110067, India,

^6^Current address: National Centre for Natural Products Research, School of Pharmacy, University of Mississippi, MS 38677, USA

*Author for correspondence

Email: [ap.sane@nbri.res.in](mailto:ap.sane@nbri.res.in); [saneanil@rediffmail.com](mailto:saneanil@rediffmail.com)

Email: [priya.willis@gmail.com](mailto:priya.willis@gmail.com)

**Supplementary tables and figures**

**Supplementary Table S1:** Summary of reads generated and mapping on rose transcriptome. (0h untreated, 8 h ethylene treated abscission zone and petal of *Rosa bourboniana*, three replicates, and 8 h ethylene treated abscission zone of *Rosa hybrida*, three replicates)

| **Sample** | **Total Reads** | **Sequence length** | **GC(%)** | **Total Data**  **(GB)** | **Alignment Reads(%)** |
| --- | --- | --- | --- | --- | --- |
| 0 h Rb-a (AZ)  0 h Rb-b (AZ)  0 h Rb-c (AZ)  8 h Rb-a (AZ)  8 h Rb-b (AZ)  8 h Rb-c (AZ)  8 h Rb-a (Petal)  8 h Rb-b (Petal)  8 h Rb-c (Petal)  8 h Rh-a (AZ)  8 h Rh-b (AZ)  8 h Rh-c (AZ) | 9385462  15214576  4511565  11408115  6871831  5294800  8111760  7865850  13429979  13600372  8412022  11693331 | 100  100  100  100  100  100  100  100  100  100  100  100 | 45  47  51  46  45  46  46  45  46  46  46  46 | 2.3  3.7  1.1  2.8  1.7  1.3  2  1.9  3.3  3.3  2.1  2.9 | 75.6  76.6  71.7  76.1  75.4  71.1  74.1  74.5  75.9  78.3  75.9  76.3 |

**Supplementary Table S6:** List of genes and primers used for study

| **S.no.** | **Genes** | **Primers**  **5'-3'** |
| --- | --- | --- |
| 1 | EIN3-binding F-box protein 1-like (RC2G0036900) | Forward:AGAACAACTCGCCAATTTGCTGAAG  Reverse: CTGTGCGAACTGACTGTATGA |
| 2 | Beta-galactosidase  (RC2G0081600) | Forward: GAGATCCCAGCAGAATTGAAGA  Reverse: GATTGCTTTGCTCTTACCACAC |
| 3 | AUX/IAA  (RC2G0452400) | Forward: GGACAGAGATGGAGATTGGA  Reverse: TAGAAGGCTTTGTCTTTCAGC |
| 4 | Protein NRT1/ PTR FAMILY 6.4 (RC2G0534800) | Forward: GATGGTGCAGCAAATGATG  Reverse: CAAGCCATCCACCAGTTCT |
| 5 | Top of Form  Polygalacturonase (RC2G0652900) | Forward:TCTCAAGGTGGTGGCTATTC  Reverse: TTCATCATAACTTCAACTCTCATGG |
| 6 | Aquaporin-like (RC1G0470300) | Forward: CTGCTCTCACAGTCCTCAAATC  Reverse: CAGGCGGTGGCTCCTTGTAGTC |
| 7 | dCTP pyrophosphatase 1-like (RC7G0052900) | Forward: TGTCAAGAATGCTAGGAAGTA  Reverse: CACACTCTAGCTTATTCTCAT |
| 8 | Lysine histidine transporter-like 8 (RC6G0303900) | Forward: TGATTGTGTCTGGTACTTATGT Reverse: ACATCACAACCAAGAGTACGTGC |
| 9 | Endochitinase 2-like  (RC6G0304900) | Forward: CGATGTAGTATAAGCTAGTTTGCT  Reverse: AGTGAAAGTAGCTCTCAGAGGG |
| 10 | Zinc finger Protein  (RC5G0340700) | Forward: GCCTTCTTCCTCTTCTACCAAC  Reverse: GCACATCTCAGTTCTCTCATCTC |
| 11 | Amine Oxidase (RC4G0064100) | Forward: TGGAGTTCTTTGAGAGCCTTG Reverse: TAACTGCGAGAAACACCGAATA |
| 12 | Wall-associated receptor kinase (RC3G0340200) | Forward: ATTCGACCTCAAGATCCTGTTG Reverse: CATAGCAGTAGGACATAAAGT |
| 13 | RbACT (beta actin) | Forward: ATGACATGGAGAAGATCTGGCATCA  Reverse: AGCCTGGATGGCAACATACATAGC |

**Supplementary Figure S1:-** FPKM plots showing comparison of biological triplicates

**
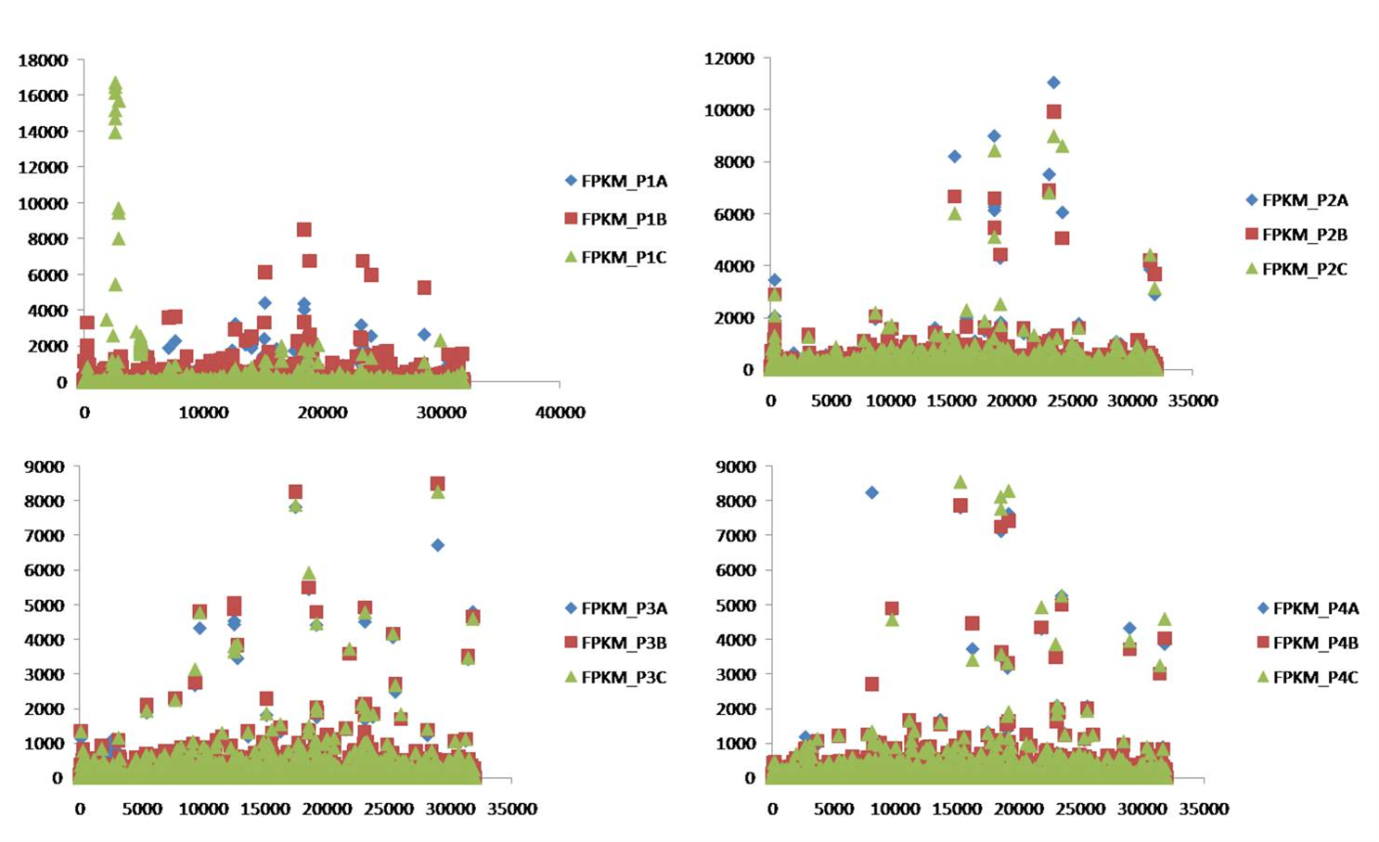
**

P1= 0 h untreated abscission zone (*Rosa bourboniana*),

P2= 8 h ethylene treated abscission zone (*Rosa bourboniana*),

P3= Petal of *Rosa bourboniana*,

P4= 8 h ethylene treated abscission zone (*Rosa hybrida*)

A, B, C = Three replicates of each sample

**Supplementary Figure S2:** Heat map of expression of few reference genes identified for qPCR; JN39925 = Elongation factor EF1α; JN39927 = Ubiquitin; JN39920 = Glyceraldehyde-3-phosphate dehydrogenase; JN39924 = serine/threonine protein phosphatase 2A ; JN39928 = SAND; JN39923 = TUBULIN, JN39921=TIP (Klie and Debener et al., 2011), KF985187=Actin (Guenin et al., 2009)

**Supplementary Figure S3. Expression profile of differential abscission-related genes related to ABA (a), Cytokinin (b), GA (c) and salicylic acid (d) pathways.** Bars represent the relative fold expression change after ethylene treatment as calculated from transcriptome data using the expression in all three biological replicates (Log2 |FC| ≤ − 1 and ≥ 1, Q-value < 0.05). Expression in respective controls was taken as one and shown as a black line across genes for comparison.

**Supplementary Figure S4.** Heat map of the 181 differentially expressed genes in different categories obtained from the comparison between 8 h ethylene-treated petal AZ and 8 h ethylene-treated petals of *R. bourboniana*. The red and green colours indicate the up and down regulation of genes, shown with + and – signs respectively in the colour bar at the top of the heat map.


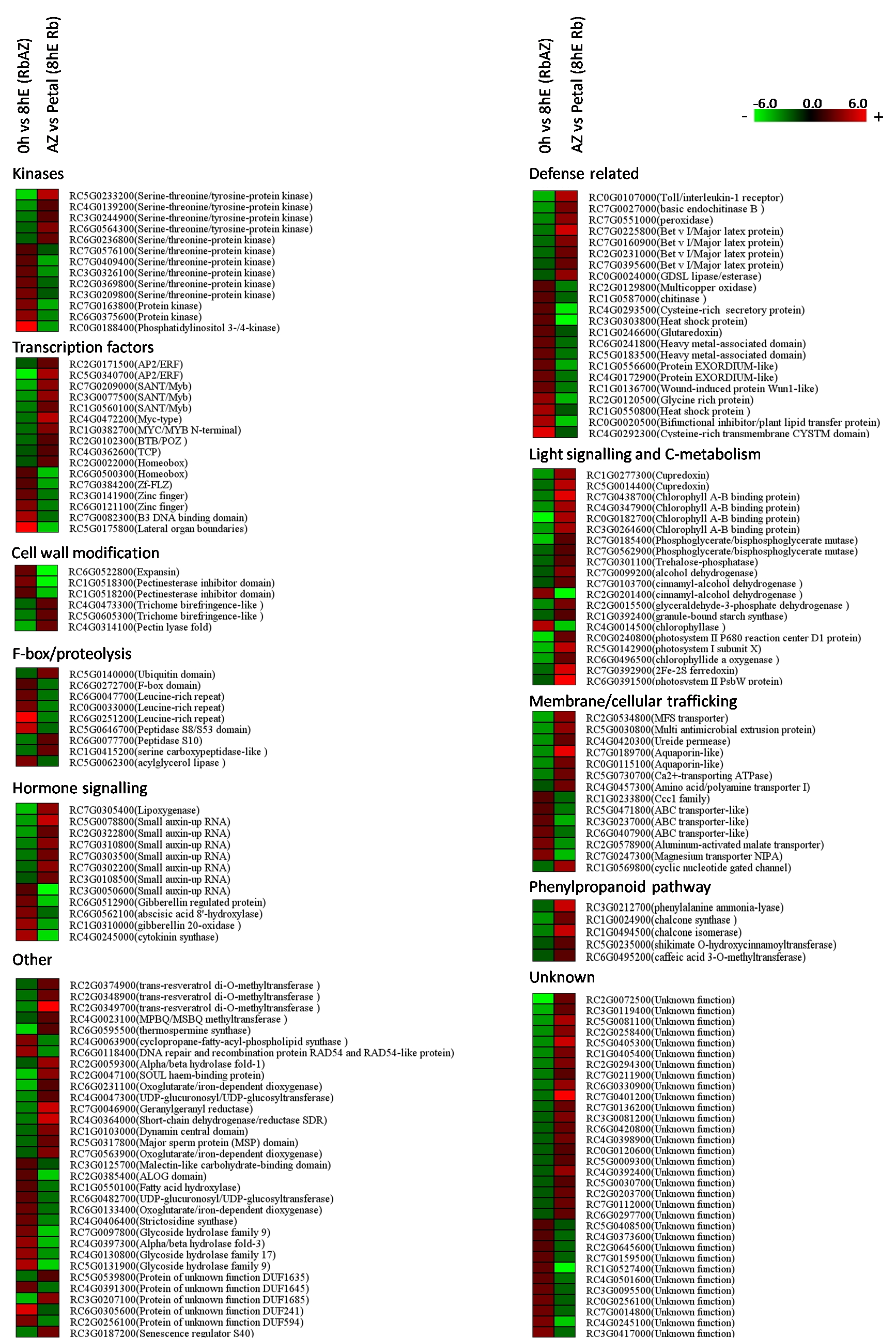


**Supplementary Figure S5.** Heat map of the 149 differentially expressed genes in different categories obtained from the comparison between 8 h ethylene-treated petal AZ of *R. bourboniana* and *R. hybrida*. The red and green colours indicate the up and down regulation of genes, shown with + and – signs respectively in the colour bar at the top of the heat map.


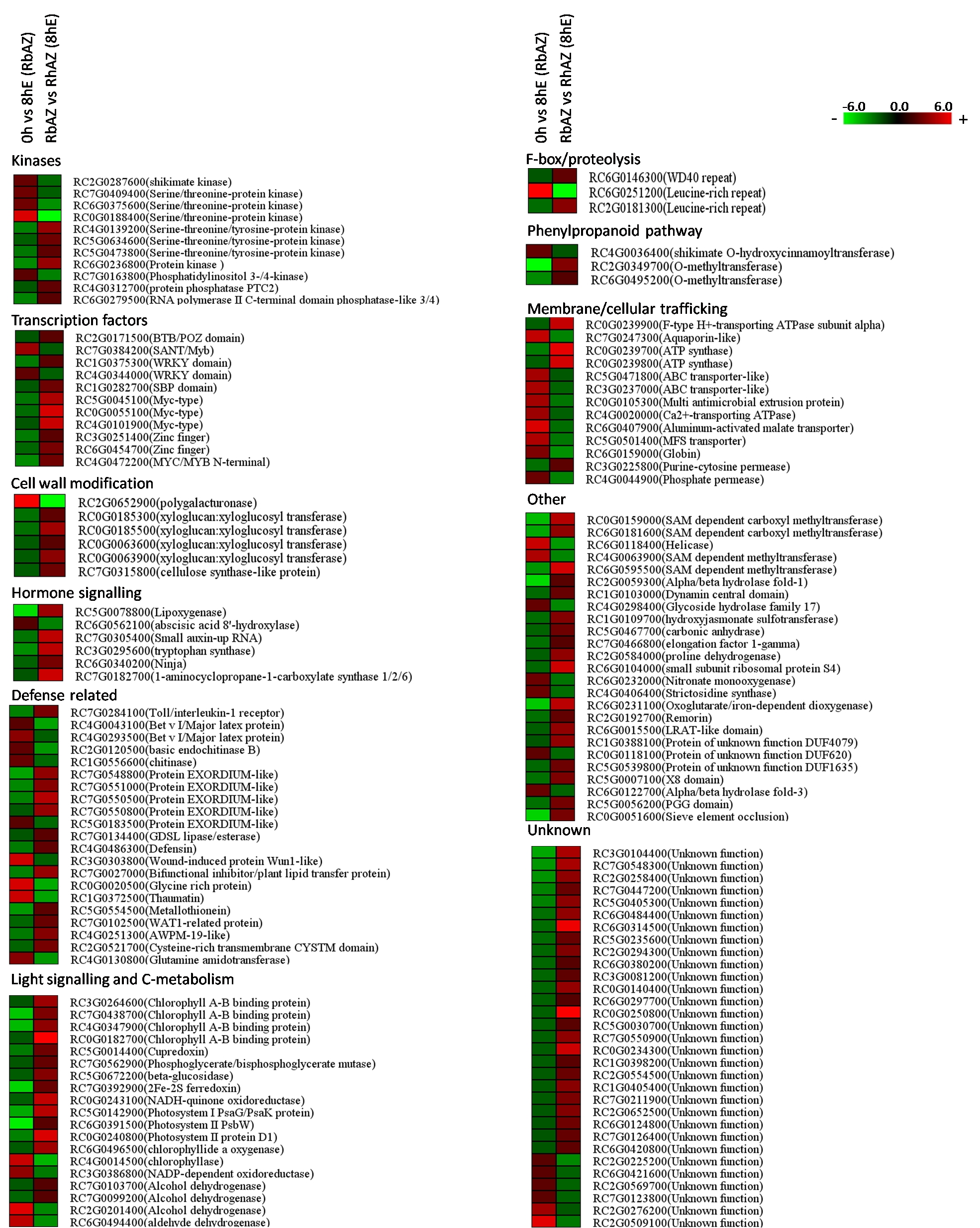

Supplement: Supplementary file 2 — Supplementary Information 2 [file 41598_2020_74144_MOESM2_ESM.docx]
